# Frequency-domain kernels enable atlas-scale detection of spatially variable genes

**DOI:** 10.64898/2026.03.12.711372

**Published:** 2026-03-19

**Authors:** Chen Yang, Xianyang Zhang, Jun Chen

**Affiliations:** 1 Department of Statistics, Texas A&M University, College Station, Texas, 77843, USA.; 2 Division of Computational Biology, Department of Quantitative Health Sciences, Mayo Clinic, Rochester, Minnesota, 55905, USA.

**Keywords:** spatially variable genes, spatial transcriptomics, random Fourier features, kernel methods, single-cell genomics

## Abstract

Identifying spatially variable genes in spatial transcriptomics requires methods that are accurate, well calibrated and scalable, yet current approaches trade expressive kernels for tractable computation. We present FLASHS, which moves spatial testing to the frequency domain: Random Fourier Features and sparse sketching enable multi-scale kernel testing on zero-inflated data without constructing distance matrices, and a kurtosis-corrected null preserves calibration. Across 50 datasets from 9 platforms, FLASHS achieves a mean Kendall τ of 0.935, exceeding the next-best method by 0.049. On the Allen Brain MERFISH atlas of 3.94 million cells, it completes in 12.6 minutes using 21.5 GB memory and maintains near-nominal false-positive rates under permutation. In human cardiac tissue, this improved ranking recovers a ventricular cardiomyocyte-associated mitochondrial biogenesis program that largely eludes parametric alternatives and replicates in an independent cohort.

## Introduction

Spatial transcriptomics technologies now measure gene expression while preserving its tissue context, enabling systematic discovery of spatially variable genes (SVGs)—genes whose expression patterns carry spatial structure beyond random fluctuation. SVGs underlie fundamental biological processes: developmental morphogen gradients that pattern embryonic tissues, metabolic zonation across the liver lobule, immune cell infiltration at tumor margins, and regionalized transcriptional programs in the brain cortex. Identifying these genes is often the first analytical step toward understanding how spatial organization shapes tissue function.

A range of computational methods have been developed for SVG detection, differing in their statistical models and scalability. Gaussian process (GP)-based approaches, including SpatialDE[1], SPARK[2], and their successors SpatialDE2[3] and nnSVG[4], provide principled likelihood-ratio or nearest-neighbor GP tests but require construction of n×n covariance matrices or per-gene iterative optimization, resulting in $O\left(n^{3}\right)$ or $O\left(n^{2}\right)$ scaling. SPARK-X[5] introduced covariance projection to reduce complexity to O(nK), substantially improving scalability, though at some cost to accuracy. Non-parametric alternatives include Moran’s I, a classical spatial autocorrelation statistic, scBSP[6], which uses granularity-based testing for efficient large-scale analysis, and Hotspot[7], which identifies informative gene modules via local autocorrelation. More recently, PreTSA[8] takes a parametric regression approach, fitting B-spline tensor product surfaces to spatial expression and testing significance via the F-statistic; this yields fast computation but constrains detection to patterns within the span of the chosen basis. Underlying these trade-offs is a common constraint: the universal kernels (Gaussian, Matérn) that detect the broadest class of spatial patterns require $O\left(n^{2}\right)$ covariance matrix construction, while scalable methods achieve their efficiency by restricting to low-rank periodic projections (SPARK-X) or fixed polynomial bases (PreTSA). Despite this progress, no existing method simultaneously achieves high detection accuracy, computational scalability to million-cell datasets, and robustness to the extreme zero-inflation characteristic of spatial transcriptomics data.

Zero-inflation is a pervasive feature of spatial transcriptomics: sequencing-based platforms such as Visium and Slide-seq routinely produce data with 80–95% zero entries per gene, reflecting both biological absence of expression and technical dropout. Most SVG detection methods operate on normalized or log-transformed data without explicitly modeling the zero component, potentially obscuring spatial patterns encoded in the presence–absence structure. Furthermore, library-size normalization can attenuate genuine spatial signal when total counts covary with tissue position, as observed along tissue gradients where cell density or capture efficiency varies spatially.

Our key insight is that the kernel–scalability trade-off can be resolved by reformulating spatial testing in the frequency domain. By Bochner’s theorem, shift-invariant spatial kernels decompose into spectral components that can be sampled via Random Fourier Features (RFF)[9], transforming kernel evaluations into inner products in a compact spectral space. Crucially, this makes the Gaussian kernel—whose universal approximation capacity is ideal for detecting arbitrary spatial patterns—computationally tractable without constructing any n×n matrix. The frequency-domain reformulation further offers three intrinsic advantages for spatial transcriptomics: multi-scale patterns map onto distinct frequency bands and are captured simultaneously; expression sparsity translates directly into computational savings, as spectral projections operate only on non-zero entries; and per-gene complexity scales with the number of expressed entries rather than total cell count.

Building on this foundation, we incorporate two additional elements for robustness. First, to handle the extreme zero-inflation of spatial transcriptomics data, a three-part test separately probes binary (presence/absence), rank (intensity order), and raw-count (absolute abundance) aspects of spatial expression, targeting complementary information that no single channel captures. Second, because spatial patterns span multiple characteristic scales—from cellular neighborhoods to tissue-wide gradients—per-scale p-values are combined via a Cauchy combination rule[10], which aggregates evidence across bandwidth scales and test types without requiring knowledge of their dependency structure.

We present FLASHS (**F**requency-domain **L**arge-scale **A**nalysis of **S**patial **H**eterogeneity), a framework implementing these ideas. On the Open Problems SVG benchmark comprising 50 datasets across 9 spatial transcriptomics platforms[11], FLASHS achieves a mean Kendall τ of 0.935, surpassing the previous best method (SPARK-X, τ=0.886) by Δτ=0.049, while maintaining memory usage below 11 GB even at one million cells. Applied to an atlas-scale MERFISH dataset of 3.94 million cells spanning the whole mouse brain, FLASHS completes in under 13 minutes with 21.5 GB memory, correctly identifies all known spatial marker genes, and provides finer-grained gene rankings than competing methods that complete at this scale. We show that the accuracy gap between FLASHS and parametric alternatives has concrete implications for biological interpretation: in human cardiac tissue, FLASHS detects 40 of 49 genes from a coherent PGC-1*α*-regulated mitochondrial biogenesis program whose spatial signal is associated with ventricular cardiomyocytes, whereas the leading parametric method detects only 1 of 49, a finding replicated in an independent cohort.

## Results

### **Overview of** FLASHS

**Fig. 1:**
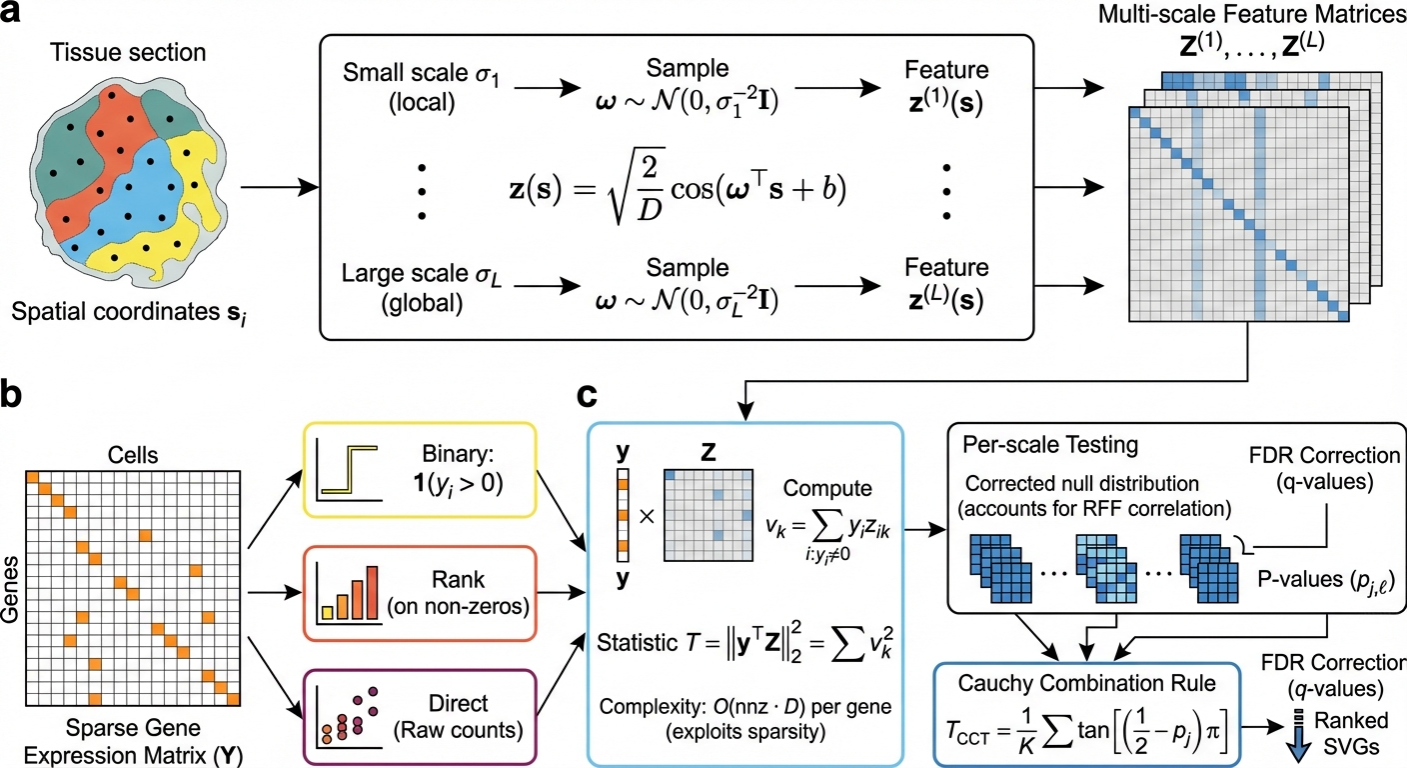
**Overview of** FLASHS. **a**, Spatial coordinates are transformed into Random Fourier Features (RFF), approximating kernel evaluations via inner products in a low-dimensional feature space. **b**, A three-part test evaluates binary expression, rank-transformed intensities, and raw counts against spatial features at multiple bandwidth scales. **c**, The test statistic $T={\left\|Z^{⊤}y\right\|}^{2}$ aggregates squared projections of expression onto spatial features. Per-scale p-values are combined via the Cauchy combination rule to yield a single p-value per gene.

For each gene g, FLASHS tests whether expression depends on spatial location $\left(H_{1}\right)$ or is spatially independent $\left(H_{0}\right)$, using Random Fourier Features (RFF) to make this test computationally tractable (Fig. 1). For each spatial location $s_{i}$, RFF constructs a D-dimensional feature vector $z\left(s_{i}\right)\in R^{D}$ whose inner products approximate a Gaussian kernel, converting kernel-based testing into linear operations. The test statistic $T={\left\|y^{⊤}Z\right\|}^{2}$ measures the total squared correlation between the expression vector $y\in R^{n}$ and the spatial feature matrix $Z\in R^{n\times D}$: under $H_{0}$, the centered projection $y^{⊤}Z$ is a sum of mean-zero terms and T is small; under $H_{1}$, spatial structure concentrates the projection onto specific frequency components, inflating T. To handle the extreme zero-inflation of spatial transcriptomics, a three-part test evaluates binary expression patterns, rank-transformed intensities, and raw counts, capturing complementary spatial signals. Per-scale p-values from all test components are merged via a Cauchy combination rule across multiple bandwidth scales, yielding a single p-value per gene.

A central algorithmic contribution is the use of sparse sketching to exploit expression sparsity. Computing $T={\left\|y^{⊤}Z\right\|}^{2}$ requires projecting y onto Z, but this projection only needs to access the non-zero entries of y. Centering, which is required for valid kernel testing, would normally destroy sparsity; FLASHS avoids this by pre-computing the column sums $1^{⊤}Z$ once and subtracting $\overset{‾}{y}\left(1^{⊤}Z\right)$, where $\overset{‾}{y}=n^{-1}\sum_{i}\;y_{i}$ is the mean expression, from the sparse projection, extending the implicit centering idea introduced by SPARK-X[5] from rank-2 cosine kernels to the *D*-dimensional RFF setting (Supplementary Note 2). This implicit centering preserves the O(nnz⋅D) pergene complexity, where nnz is the number of non-zero entries—typically 5–20% of the total cell count in spatial transcriptomics data.

FLASHS has two main tuning parameters: the number of RFF features D (default 500) and the number of bandwidth scales L (default 7). The bandwidths $\sigma_{1},\ldots ,\sigma_{L}$ are selected adaptively from data-intrinsic length scales rather than specified by the user (Methods). Since the total feature count D is fixed, changing L adjusts scale resolution without affecting per-gene cost, which remains O(nnz⋅D). Sensitivity analysis confirms that the defaults provide a robust operating point across all 50 benchmark datasets (Supplementary Fig. 1a–d).

### Benchmark accuracy on the Open Problems SVG evaluation

We evaluated FLASHS on the Open Problems benchmark for SVG detection[11], a standardized evaluation framework comprising 50 datasets across 9 spatial transcriptomics platforms (Visium, Slide-seq V2, MERFISH, STARmap, Stereo-seq, seqFISH, DBiT-seq, Slide-tags, and Post-Xenium). Datasets span diverse organisms (human, mouse, *Drosophila*) and tissues (brain, cancer, embryo, heart, kidney, and others), with sizes ranging from 936 to 29,309 spots and 210 to 1,050 genes per dataset. Ground truth spatial variability scores were generated using scDesign3[12] by mixing Gaussian process and non-spatial models. Performance is measured by Kendall *τ* correlation between predicted and true spatial variability scores, averaged across genes grouped by their original feature identity.

**Fig. 2:**
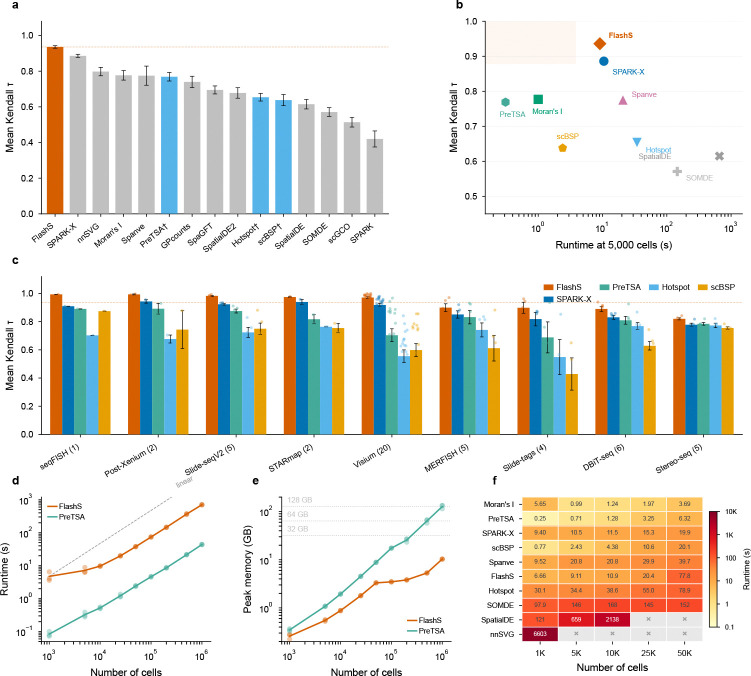
Benchmark accuracy, scalability, and per-platform performance. **a**, Mean Kendall τ correlation between predicted and ground-truth spatial variability scores across 50 datasets spanning 9 platforms. FLASHS (red) achieves τ=0.935, surpassing SPARK-X (τ=0.886) by Δτ=0.049. PreTSA, Hotspot and scBSP (marked with †) were benchmarked independently on the same datasets. Error bars indicate standard error of the mean. **b**, Accuracy versus runtime (at 5,000 cells). FLASHS occupies the Pareto-optimal region of high accuracy and fast runtime (shaded). **c**, Per-platform mean Kendall τ for FLASHS, SPARK-X, PreTSA, Hotspot, and scBSP across 9 platforms, sorted by FLASHS performance. Individual dataset values shown as dots. **d**, Runtime as a function of cell number (5,000 genes). Both FLASHS and PreTSA scale near-linearly; dashed line indicates linear scaling. **e**, Peak memory usage. PreTSA’s memory scales as O(n⋅g), crossing 32 GB at ~200,000 cells and reaching 137 GB at 1,000,000 cells; FLASHS remains below 11 GB via sparse sketching. Dotted lines mark typical workstation memory. Points show individual replicates (n=3 per size). **f**, Runtime heatmap for 10 SVG detection methods across cell counts (1,000–50,000), sorted by speed at 50,000 cells. Cell values show mean runtime in seconds; × marks timeout (>2 h) or out-of-memory failure. All benchmarks performed on a 16-core compute node.

FLASHS achieves a mean Kendall τ of 0.935 across all 50 datasets, establishing a new state of the art (Fig. 2; Supplementary Table 1). This surpasses the previous best method, SPARK-X (τ=0.886), by Δτ=0.049. We independently validated this comparison by running SPARK-X in the same computing environment on all 50 datasets, obtaining τ=0.881 (consistent with the leaderboard value), with FLASHS outperforming on every dataset (paired sign test, $p=2^{-50}<10^{-15}$; Δτ=+0.054; Supplementary Table 10). Notably, FLASHS maintains positive correlation across all 50 datasets (minimum τ=0.788), whereas several competing methods produce negative correlations on individual datasets, indicating anti-correlated predictions. Performance is consistent across all 9 platforms, with mean τ>0.80 on every platform (Supplementary Fig. 2).

To contextualize the accuracy–scalability trade-off, we additionally benchmarked three scalable methods not included in the original Open Problems leaderboard—PreTSA[8], Hotspot[7], and scBSP[6]—on the same 50 datasets (Fig. 2a). PreTSA, which applies B-spline tensor product regression with an F-test, achieves mean τ=0.769; Hotspot achieves mean τ=0.654 and scBSP mean τ=0.638. Despite its computational efficiency, PreTSA’s parametric regression captures smooth spatial trends but cannot model the multi-scale, non-smooth patterns that kernel-based methods detect, resulting in a Δτ=0.167 gap relative to FLASHS. On the accuracy–runtime Pareto frontier (Fig. 2b), FLASHS is more accurate than other scalable alternatives and faster than methods of comparable accuracy.

### Computational scalability

We benchmarked FLASHS runtime and memory usage on simulated datasets with 5,000 genes and cell counts ranging from 1,000 to 1,000,000 (Fig. 2d,e). Runtime scales near-linearly with cell number: from 4.7 seconds at 1,000 cells to 74 seconds at 100,000 cells and 703 seconds (11.7 minutes) at 1,000,000 cells (3 replicates per size). Peak memory usage reaches 10.3 GB at 1,000,000 cells, dominated by the input expression matrix in sparse format; the n×D feature matrix Z is never materialized (Methods). These benchmarks used default parameters (D=500 features, L=7 scales) on a 16-core compute node (Supplementary Table 2).

To contextualize these results, we compared FLASHS runtime against nine established SVG detection methods on the same simulated data (Fig. 2f; Supplementary Table 3): Moran’s I (via Squidpy[13]), SpatialDE[1], Hotspot[7], SPARK-X[5], SOMDE[14], scBSP[6], Spanve[15], nnSVG[4], and PreTSA[8].

PreTSA achieves the lowest wall-clock time at all tested scales through shared hat-matrix dense operations, but its memory scales as O(n⋅g), reaching 137 GB at one million cells—13-fold more than FLASHS’s 10.3 GB (Supplementary Table 4). On a typical workstation (32–64 GB RAM), PreTSA becomes infeasible beyond ~200,000 cells, whereas FLASHS handles one million cells within 16 GB. scBSP, Moran’s I, and SPARK-X offer comparable or faster runtimes but achieve lower accuracy (Fig. 2a; Supplementary Table 3).

GP-based methods are orders of magnitude slower. SpatialDE requires 659 seconds at 5,000 cells and exceeds the 2-hour timeout at ≥25,000 cells. nnSVG, which fits a nearest-neighbor GP per gene with iterative REML, requires ~6,600 seconds (~110 minutes) at just 1,000 cells with 5,000 genes and exceeds the 2-hour timeout at all larger cell counts (5,000–50,000). Among methods with competitive accuracy (τ>0.85), FLASHS is the fastest.

### Statistical calibration under the null

To assess p-value calibration, we generated null datasets on 2,500-spot grids at three zero-fraction levels (~50%, ~90%, and ~95%) with expression drawn from negative binomial distributions without spatial structure (20 replicates per sparsity level, 1,000 genes each). Two technical challenges arise: (i) the RFF columns $z_{k}$ are correlated, particularly at large bandwidth scales, and (ii) zero-inflated expression vectors are leptokurtic, violating the Gaussian assumption of the standard Satterthwaite approximation. We derive a kurtosis-corrected moment-matching formula that accounts for both the per-scale covariance structure among RFF features and the excess kurtosis of the expression distribution (Methods; Supplementary Note 3; Supplementary Fig. 4).

**Fig. 3:**
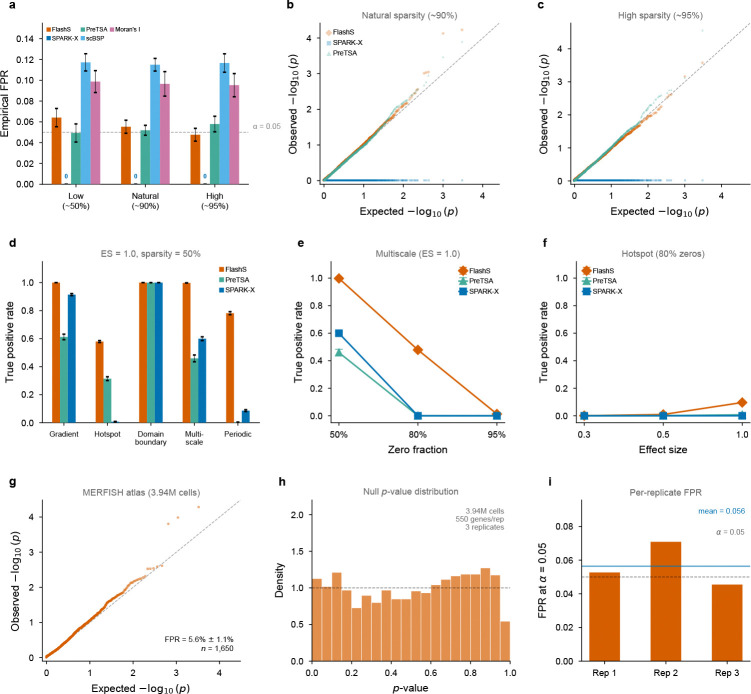
Statistical calibration and detection power. **a**, Empirical false positive rates at α=0.05 across five methods and three sparsity levels (~50%, ~90%, ~95% zeros; 20 replicates of 1,000 genes each). Dashed line marks the nominal level. SPARK-X yields FPR = 0 in all conditions (annotated). **b,c**, QQ plots of null p-values at natural (~90%) and high (~95%) sparsity (simulation). FLASHS and PreTSA track the diagonal closely; SPARK-X p-values concentrate at 1 (strongly conservative). **d**, Cross-method detection power (TPR at q<0.05) across five spatial pattern types at effect size 1.0 and 50% sparsity. FLASHS achieves the highest sensitivity across all pattern types; SPARK-X shows near-zero power on hotspot patterns. **e**, TPR as a function of sparsity for the multiscale pattern (effect size 1.0). At 80% zeros, FLASHS retains substantial power while SPARK-X and PreTSA drop near zero. **f**, TPR as a function of effect size for the hotspot pattern at 80% sparsity. **g**, QQ plot of null p-values from global row permutation on the Allen MERFISH atlas (3.94 million cells, 550 genes, 3 replicates pooled, 1,650 null tests). FLASHS maintains near-nominal FPR at million-cell scale (mean FPR = 5.6% ± 1.1%). **h**, Histogram of MERFISH null p-values with uniform reference density. **i**, Per-replicate false positive rate at α=0.05 on the MERFISH atlas; mean FPR (blue line) tracks the nominal level. Error bars in **d–f** indicate standard error across 10 replicates.

With the corrected null distribution, the empirical false positive rate at α=0.05 averages 5.6% across 60 replicates (Fig. 3a; Supplementary Fig. 3b), close to the nominal level and decreasing toward the nominal 5% at higher sparsity (4.8% at ~95% zeros). Under these simulation conditions, the q-value-based false discovery rate[16] remained below 0.1% at q<0.05 across all 60 replicates. Per-scale calibration is accurate across all bandwidth scales, including large scales where RFF features are most correlated (Supplementary Fig. 10a).

To contextualize FLASHS’s calibration, we compared empirical false positive rates with SPARK-X, PreTSA, scBSP, and Moran’s I on null datasets at three zero-fraction levels (~50%, ~90%, ~95%; Fig. 3a). FLASHS (4.8–6.4%) closely tracks the nominal level, with calibration improving at higher sparsity where the kurtosis correction is most impactful. PreTSA (4.9–5.8%) is similarly well-calibrated overall, though its FPR increases with sparsity—consistent with increasing violation of the F-test normality assumption at higher zero fractions. SPARK-X yields FPR = 0 in all conditions, indicating a strongly conservative null distribution (Fig. 3b). scBSP (11.5–11.7%) and Moran’s I (9.5–9.9%) show substantially inflated Type I error, indicating inadequate accounting for zero-inflation when computing p-values under the null.

To confirm that calibration extends to atlas-scale data with realistic spatial geometry, we performed global permutation testing on the full Allen MERFISH dataset (3.94 million cells, 550 genes). Global row permutation—shuffling which cell receives which expression vector across all cells—destroys all spatial structure while preserving the real coordinate layout and per-gene marginal distributions. Across three independent replicates (1,650 total null tests), FLASHS maintained a mean FPR of 5.6% ± 1.1% at α=0.05 (Fig. 3g–i), confirming calibrated inference at million-cell scale on real spatial coordinates.

A natural concern is whether FLASHS’s detections reflect genuine spatial structure or method miscalibration. We addressed this using the exchangeability-block framework[17] with two structured permutation schemes that selectively preserve part of the spatial organization: (i) within-section permutation, which shuffles expression only among cells within the same brain section, preserving between-section spatial structure; and (ii) spatial block permutation, which partitions each section into a 6×6 grid and permutes expression vectors between grid blocks, preserving within-block spatial autocorrelation. Under both schemes, FLASHS rejects 100% of genes across all replicates (Supplementary Table 9)—an expected result, because these permutations retain substantial spatial organization at scales detectable by the multi-scale kernel. These structured controls are therefore not calibration tests; rather, they show that when part of the biological structure is retained, FLASHS continues to detect it. Interpreted alongside the global null (5.6% FPR), this pattern supports the view that detections are driven by real spatial organization of gene expression rather than indiscriminate rejections.

### Detection power across spatial pattern types and sparsity levels

We evaluated detection power across three spatial pattern types—hotspot (localized expression), gradient (tissue-wide trend), and periodic (oscillatory)—at varying effect sizes and sparsity levels (Supplementary Fig. 3c). The false discovery rate is controlled near the nominal 5% level across all conditions. Gradient patterns are detected with near-perfect power (TPR = 95.5–100%) even at high sparsity, confirming that the multi-scale RFF effectively captures tissue-wide trends. Hotspot patterns achieve strong detection at low sparsity (TPR > 93%) with power decreasing at high sparsity (TPR ≈ 47% at 50% zero fraction), consistent with the challenge of detecting localized signals when most observations are zero. Periodic patterns show higher variance across replicates and lower mean TPR at moderate effect sizes (5–35% at effect sizes 0.1–0.5); at stronger effect sizes the multi-scale bandwidth ensemble achieves 78% TPR, exceeding hotspot detection (58%) and all competitor methods (Fig. 3d).

Sensitivity analysis on the two key hyperparameters—number of random features D and bandwidth scales L—confirms that the default settings (D=500,L=7) provide a robust operating point: power stabilizes above D≈500 and requires multiple bandwidth scales (L≥5) for reliable multi-scale detection (Supplementary Fig. 1a–d).

To compare detection profiles across methodological paradigms, we simulated five canonical spatial pattern types—gradient, hotspot, domain boundary, multiscale (gradient + hotspot), and periodic (sinusoidal)—with matched effect sizes and evaluated FLASHS, SPARK-X, and PreTSA (Fig. 3d). At effect size 1.0 and 50% sparsity, FLASHS achieves the highest sensitivity across all five pattern types. The advantage is largest for hotspot patterns, where SPARK-X shows near-zero power (TPR = 0.7%) consistent with its reliance on periodic covariance kernels that are poorly suited to focal spatial signals. On periodic patterns, FLASHS achieves TPR = 78.2%, far exceeding both SPARK-X (8.6%) and PreTSA (0.3%)—notably, SPARK-X’s periodic cosine kernels do not confer an advantage because their fixed frequency spacing does not match the simulated signal frequency. At higher sparsity (80% zeros), FLASHS retains substantial power on multiscale patterns where both SPARK-X and PreTSA drop below 1% (Fig. 3e). These detection gaps on compound and non-smooth patterns predict that parametric methods will systematically miss genes with multi-scale spatial structure in real tissue—a prediction confirmed by the cardiac tissue analysis (Fig. 5).

### Cross-dataset and cross-platform generalization

**Fig. 4:**
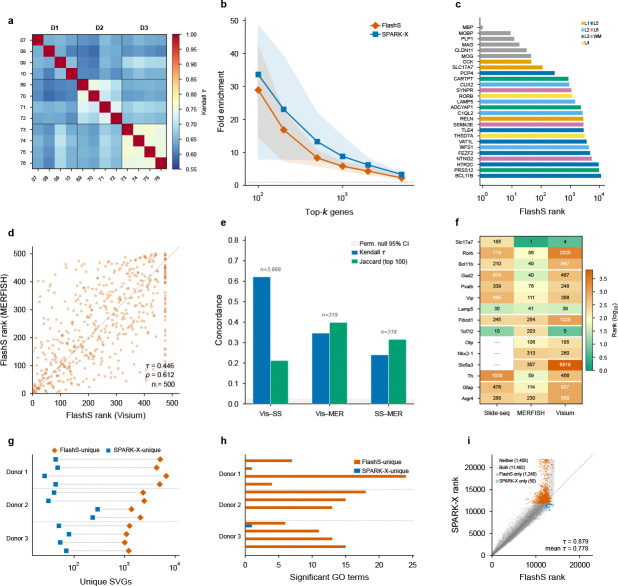
Cross-dataset and cross-platform generalization of SVG rankings. **a**, Cross-sample reproducibility of FLASHS SVG rankings across 12 DLPFC Visium samples (3 donors). Same-donor pairs (diagonal blocks) show higher concordance (mean τ=0.703) than cross-donor pairs (mean τ=0.643). **b**, Fold enrichment of 27 known cortical layer markers in the top-*k* ranked genes, averaged across all 12 samples. Both FLASHS (vermillion) and SPARK-X (blue) strongly enrich known markers. **c**, FLASHS ranks of all 27 known cortical layer markers (Maynard et al. 2021), colored by assigned cortical layer. White-matter markers (MBP, MOBP, PLP1) are ranked highest, followed by layer-specific markers in order of their spatial variability. All 27 markers are detected as SVGs (q<0.05). **d**, Cross-platform SVG rank concordance between Visium mouse brain (2,702 spots) and MERFISH atlas (3.94 million cells) for 500 common genes. FLASHS rankings show significant concordance (Kendall τ=0.446, Spearman ρ=0.612, permutation P<0.001). **e**, Pairwise rank concordance (Kendall τ, blue) and top-100 gene overlap (Jaccard similarity, green) across three spatial transcriptomics platforms. All pairs significantly exceed the permutation null (grey band, 95% CI). Concordance is strongest between sequencing-based platforms (Visium–Slide-seq, τ=0.605,3,666 genes) and moderate for cross-modality comparisons involving the targeted MERFISH panel (319 genes). n, number of common genes tested. **f**, Rankings of 15 established brain region markers across three platforms. Each row is a gene; columns show the rank (lower = stronger spatial signal) on Slide-seq V2, MERFISH, and Visium. Known markers are consistently ranked among the top SVGs across all three technologies. **g**, Method-specific SVG counts across all 12 DLPFC samples (log scale). Restricting to commonly tested genes with Benjamini–Hochberg correction at q<0.05, FLASHS consistently detects substantially more unique SVGs (mean 2,802; vermillion) than SPARK-X (mean 84; blue). Lines connect paired counts for each sample. **h**, Number of significant Gene Ontology enrichment terms for method-specific SVGs across all 12 DLPFC samples. FLASHS-unique SVGs yield 0–24 enriched terms per sample; SPARK-X-unique SVGs yield zero terms in 11 of 12 samples. **i**, Rank scatter plot comparing FLASHS and SPARK-X on DLPFC sample 151673 (14,248 commonly tested genes). After Benjamini–Hochberg correction at q<0.05, FLASHS detects substantially more unique SVGs than SPARK-X, with both methods showing highly concordant rankings overall.

To assess cross-sample reproducibility, we applied FLASHS to 12 Visium samples spanning the human dorsolateral prefrontal cortex (3 donors, 33,538 genes; Maynard et al.[18]). SVG rankings show high concordance across biological replicates (same-donor mean τ=0.703, cross-donor mean τ=0.643; Fig. 4a), with expected inter-individual variation exceeding technical noise. All 27 known cortical layer markers were detected as SVGs (q<0.05), with white-matter markers (MBP, MOBP, PLP1) ranked highest, consistent with the expected depth gradient (Fig. 4c; Supplementary Fig. 8). Running SPARK-X independently on all 12 samples yielded a mean cross-method rank concordance of τ=0.778, with higher agreement within Donor 3 (τ=0.88) than Donor 1 (τ=0.62), reflecting biologically meaningful inter-individual variation in spatial transcriptomic patterns. Across the 12-sample average, both methods strongly enrich known cortical markers (top-100: FLASHS 29.0× vs SPARK-X 33.7×; Fig. 4b,c), confirming reproducible and biologically coherent gene rankings.

As a stringent cross-platform test, we compared SVG rankings produced independently on two mouse brain datasets profiled by different technologies: a 10x Visium adult mouse brain dataset (2,702 spots, full transcriptome) and the Allen MERFISH atlas (3.94 million cells, 550 targeted genes). Among 500 genes tested on both platforms, FLASHS rankings show significant cross-platform concordance (Kendall τ=0.446, Spearman ρ=0.612, permutation P<0.001; Fig. 4d), comparable to PreTSA (τ=0.460,ρ=0.639) and far exceeding the permutation null ($\tau_{\text{null}}=0.000\pm 0.029$). Top-gene overlap confirms this concordance, with both methods substantially exceeding chance at all thresholds (Jaccard@100: FLASHS = 0.41, PreTSA = 0.42, null = 0.11; Supplementary Fig. 6a). Known brain region markers—including cortical excitatory/inhibitory, hippocampal, astrocytic, and oligodendrocyte markers—are consistently ranked among the top SVGs on both platforms (Supplementary Fig. 6b), indicating that the detected spatial signals reflect genuine biology that replicates across technologies despite a 1,400-fold difference in cell count and fundamentally different measurement modalities. Extending to a third technology, Slide-seq V2 (41,786 beads, hippocampus), SVG rankings show strong concordance with Visium (Kendall τ=0.605,3,666 genes) and moderate concordance with MERFISH (τ=0.200,319 genes; Fig. 4e). All three pairwise comparisons significantly exceed the permutation null (P<0.001), with concordance scaling with gene overlap and platform similarity (Fig. 4f; Supplementary Fig. 6d,e).

Beyond quantitative concordance, FLASHS and SPARK-X show a striking asymmetry in unique detections: restricting to commonly tested genes with Benjamini–Hochberg correction at q<0.05, FLASHS detects a mean of 2,802 unique SVGs across the 12 samples versus only 84 for SPARK-X (Fig. 4g). Gene Ontology enrichment analysis reveals that this asymmetry carries biological significance: FLASHS-unique SVGs converge on DNA-binding transcription factor activity as the top enriched term in 7 of 12 samples (e.g., $p=9.2\times 10^{-5}$ in sample 151673), consistent with the spatial organization of cortical layer-specific transcriptional programs (Fig. 4h; Supplementary Table 8). By contrast, SPARK-X-unique SVGs yield zero enriched GO terms in 11 of 12 samples, with the sole exception being a single marginally enriched term (C-type lectin signaling) unrelated to cortical architecture.

### Biological validation and pathway-level coherence in cardiac tissue

**Fig. 5:**
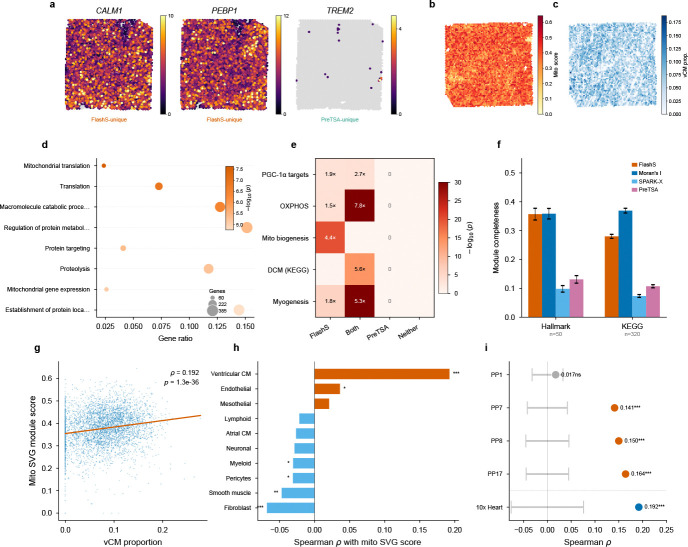
Biological validation and pathway-level coherence in human heart. **a**, Spatial expression of discordant SVGs on the Visium human heart (4,235 spots): *CALM1* (calmodulin 1) and *PEBP1* (phosphatidylethanolamine-binding protein 1; FLASHS-unique, clear spatial patterns across cardiac tissue) versus *TREM2* (triggering receptor expressed on myeloid cells 2; PreTSA-unique, sparse expression in myeloid-enriched foci). Color intensity indicates raw counts; gray dots mark non-expressing spots. Full discordant gene comparison in Supplementary Fig. 7. **b**, Spatial distribution of the mitochondrial SVG module score (mean log-normalized expression of 49 curated mitochondrial biogenesis genes) across 4,235 Visium spots. **c**, Spatial distribution of deconvolved ventricular cardiomyocyte (vCM) proportion across the tissue, estimated by FlashDeconv using the Litviňuková et al. single-cell atlas. **d**, Gene Ontology enrichment of 2,669 FLASHS-unique SVGs, showing spatially organized processes (mitochondrial translation, intracellular transport) missed by PreTSA. **e**, Enrichment heatmap showing hypergeometric significance (−log_10_(*p*)) of four SVG categories across five curated gene sets (MSigDB). Annotations indicate fold enrichment for significant results (p<0.05); all marked results remain significant after Benjamini–Hochberg correction (q<0.05; Supplementary Table 7). FLASHS-unique SVGs are strongly enriched for mitochondrial pathways; PreTSA-unique SVGs show no significant overlap with any gene set. **f**, Systematic module completeness across 365 curated pathways (50 MSigDB Hallmark, 315 KEGG) within the 14,634-gene universe. Bars show mean fraction of pathway genes detected as SVGs (q<0.05) by each method; error bars indicate s.e.m. FLASHS achieves the highest completeness (mean 0.363), surpassing Moran’s I (0.310) and outperforming parametric alternatives by 2.6–3.7×. Per-pathway distributions and method-specific SVG counts in Supplementary Fig. 9. **g**, Scatter plot of vCM proportion versus mitochondrial SVG module score ($\rho =0.192,p=1.3\times 10^{-36}$). **h**, Spearman correlation of mitochondrial SVG module score with each deconvolved cell-type proportion. Ventricular cardiomyocytes show the clearest positive correlation, consistent with their high mitochondrial content. Block-permutation and gene-set leakage controls are shown in Supplementary Fig. 7. **i**, External replication on four independent healthy control Visium slides from Kuppe et al.[24] (2,043–4,269 spots per slide). Points show Spearman ρ between the transferred mito module score and cell2location-estimated cardiomyocyte proportion; gray bands show block-permutation null 95% CI. Three of four replication samples show significant positive association $\left(\rho =0.14-0.16,p_{\text{perm}}<10^{-4}\right)$. Stars: Spearman $p\;{(}^{***}p<0.001$).

The simulation analyses predict that parametric methods will systematically miss genes whose spatial patterns span multiple scales. To test whether this translates to biologically meaningful detection differences, we compared SVG detections by FLASHS, SPARK-X, and PreTSA on a 10x Visium human heart dataset (4,235 spots, 16,716 genes; Fig. 5a). FLASHS and SPARK-X show the highest pairwise rank agreement (Kendall τ=0.52, top-50 overlap 80%), consistent with both being non-parametric kernel-based approaches; both show lower agreement with PreTSA (τ=0.41 and 0.38, respectively), confirming that the parametric B-spline approach produces substantially different gene rankings. Restricting to genes tested by both methods and applying Benjamini–Hochberg correction uniformly, FLASHS detects 3,699 SVGs at q<0.05 compared to 1,123 for PreTSA, with 1,030 genes detected by both methods and 93 detected only by PreTSA. Gene Ontology enrichment analysis reveals that the shared SVGs are strongly enriched for cardiac-specific functions, led by oxidative phosphorylation $\left(p<10^{-72}\right)$ and aerobic respiration, consistent with the spatial organization of energy metabolism in heart tissue. FLASHS-unique SVGs (2,669 genes) are enriched for additional spatially organized processes including mitochondrial translation and intracellular transport (Fig. 5d; Supplementary Table 5). In contrast, the 93 PreTSA-unique SVGs yield no significant GO terms, suggesting that these detections may be model-specific and lack support from known biological pathways. Visual inspection confirms this asymmetry: FLASHS-unique SVGs such as *CALM1* and *PEBP1* show clear spatial expression patterns across the cardiac tissue, whereas top PreTSA-unique detections (e.g., *TREM2*) exhibit sparse expression limited to isolated foci (Fig. 5a; Supplementary Fig. 7).

We next asked whether this enrichment reflects a coherent biological program and whether the detected spatial patterns can be independently validated using orthogonal single-cell data.

To test this, we performed hypergeometric enrichment analysis against five curated gene sets from MSigDB[19], applying Benjamini–Hochberg correction across all 20 tests (5 gene sets × 4 SVG categories; Supplementary Table 7). FLASHS-unique SVGs are significantly enriched for PGC-1*α* transcriptional targets (1.90-fold, odds ratio [OR] = 2.39, 95% CI 1.51–3.83, $q=1.0\times 10^{-3}$; Fig. 5e), the master regulator of mitochondrial biogenesis in cardiomyocytes[20, 21]. The dataset contains 49 curated mitochondrial biogenesis genes, spanning mitochondrial ribosomal proteins (MRPL/MRPS family), translocase complexes (TOMM/TIMM), and respiratory chain assembly factors. Of these, 40 are detected as SVGs by FLASHS—39 appearing exclusively in the FLASHS-only category (4.36-fold, $q=9.5\times 10^{-20}$)—with no overlap in the PreTSA-only category. This sensitivity advantage extends beyond the parametric–non-parametric divide. Moran’s I, a standard global autocorrelation statistic, detects only 28 of 49 (57%) of these genes at q<0.05 despite calling 51% of the transcriptome spatially variable genome-wide; SPARK-X detects only 9 (18%; Supplementary Table 11). The resulting detection gradient—FLASHS (40) > Moran’s I (28) > SPARK-X (9) > PreTSA (1)—tracks the flexibility of each method’s spatial representation. These 40 genes form a spatially coordinated transcriptional program: they share a common upstream regulator (PGC-1*α*), are co-expressed within myocardial compartments, and their collective spatial signature—focal enrichment in ventricular regions superimposed on tissue-wide metabolic gradients—exemplifies the compound, multi-scale patterns that a fixed-degree polynomial basis is not designed to capture. This enrichment extends to disease-relevant gene sets: dilated cardiomyopathy genes (KEGG; OR = 8.81, 95% CI 5.50–14.28, $q=4.6\times 10^{-14}$ in the shared SVG category) and myogenesis hallmark genes (OR = 8.32, 95% CI 6.12–11.38, $q=2.4\times 10^{-30}$) are enriched among SVGs detected by FLASHS, while PreTSA-unique SVGs show no significant overlap with any tested pathway (0 of 20 tests significant after FDR correction; Fig. 5e).

To validate the spatial pattern of these mitochondrial SVGs independently, we performed spatial deconvolution using the Litviňuková et al. human heart single-cell RNA-seq atlas[22] (452,136 cells, 12 cell types) via FlashDeconv[23]. We then computed a per-spot mitochondrial SVG module score (mean log-normalized expression of the 49 curated mitochondrial biogenesis genes) and correlated it with deconvolved cell-type proportions across 4,235 Visium spots (Fig. 5b,c). The mitochondrial SVG score is positively correlated with ventricular cardiomyocyte proportion (Spearman=0.192, $p=1.3\times 10^{-36}$; Fig. 5g), consistent with the known high mitochondrial content of ventricular cardiomyocytes, which depend on oxidative phosphorylation for continuous contractile function[22]. By contrast, atrial cardiomyocyte proportion shows no significant correlation (ρ=−0.026,p=0.085), reflecting the lower mitochondrial density in atrial tissue, while fibroblasts are negatively correlated ($\rho =-0.068,\;p=9.4\times 10^{-6}$; Fig. 5h). Among all 12 cell types tested, ventricular cardiomyocytes show the clearest positive association with the mitochondrial SVG pattern.

Two controls confirm the independence of this spatial correlation. First, spatial block permutation testing—which preserves within-block autocorrelation by shuffling tissue grid blocks rather than individual spots—confirms that the correlation is robust to spatial dependence ($p_{\text{perm}}<10^{-4}$ across three grid resolutions, 6 × 6, 8 × 8, and 10×10; null $|\rho {|}_{\text{max}}=0.076$; Supplementary Fig. 7). Second, to exclude gene-set leakage between the module score and deconvolution input, we re-ran FlashDeconv after removing all 49 mitochondrial biogenesis genes from the Visium expression matrix; the vCM correlation remains virtually unchanged (ρ=0.187, $p=1.4\times 10^{-34}$, Δρ=0.006; Supplementary Fig. 7), confirming that the observed association is not driven by shared gene features between the two measurements.

To test whether this mito–cardiomyocyte association generalizes beyond a single dataset, we repeated the full analysis on four healthy control Visium slides from an independent cohort[24] (2,043–4,269 spots per slide). Despite only 16 of the 49 mito biogenesis genes being present in the Kuppe Visium panel, FLASHS-unique SVGs are again significantly enriched for mitochondrial biogenesis in all 4 samples (mean fold enrichment = 2.8, hypergeometric p<0.02; Supplementary Fig. 7). The transferred 49-gene mito module score is positively associated with cell2location-estimated cardiomyocyte proportions in 3 of 4 samples (ρ=0.14−0.16, block permutation $p_{\text{perm}}<10^{-4}$; Fig. 5i), replicating the key finding on independent tissue from a different institution and experimental batch.

Together, these results provide convergent evidence that FLASHS recovers a coherent and reproducible biological program: (i) FLASHS detects a set of spatially variable genes that PreTSA largely misses; (ii) these genes converge on PGC-1*α*-driven mitochondrial biogenesis; (iii) single-cell deconvolution shows that the spatial signal is most strongly associated with ventricular cardiomyocytes; and (iv) this evidence chain replicates in an independent cardiac cohort. The absence of pathway enrichment among PreTSA-unique SVGs, combined with FLASHS’s near-complete capture of the mitochondrial module (Fig. 5e), indicates that this detection gap extends to the functional-module level. To assess whether the pattern generalizes beyond individual pathways, we performed a systematic module completeness analysis across 365 curated gene sets (50 MSigDB Hallmark and 315 KEGG pathways), computing for each pathway the fraction of its genes detected as SVGs by each method within the same 14,634-gene universe (Fig. 5f; Supplementary Table 12). This pattern persists at the genome-wide pathway level: FLASHS recovers a mean of 40.5% of Hallmark pathway genes as SVGs, surpassing Moran’s I (33.5%)—which calls 51% of its genome-wide transcriptome as spatially variable—while PreTSA and SPARK-X achieve markedly lower coverage (14.9% and 11.2%, respectively). Within the common gene universe, FLASHS detects more SVGs than Moran’s I (3,699 vs 3,006) and achieves 17% higher module completeness (mean 0.363 vs 0.310 across all pathways), indicating that its multi-kernel approach has genuine selectivity for pathway-relevant spatial patterns. A top-*k* controlled analysis—comparing methods at the same gene count cutoff rather than method-specific q-value thresholds—confirms that FLASHS’s pathway coverage advantage persists after removing the confound of differing SVG counts (Supplementary Fig. 10c).

### Atlas-scale application to whole mouse brain MERFISH

**Fig. 6:**
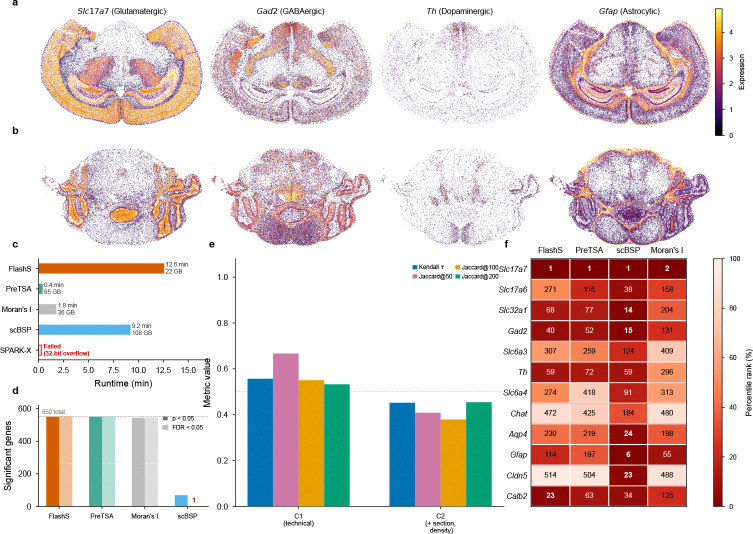
Atlas-scale SVG detection on the Allen MERFISH whole mouse brain. **a,b**, Spatial expression of four representative marker genes in mid-brain (**a**, 115,779 cells) and anterior (**b**) coronal sections: *Slc17a7* (glutamatergic, cortex), *Gad2* (GABAergic), *Th* (dopaminergic, midbrain), *Gfap* (astrocytic). Color intensity indicates log-transformed expression; gray dots mark non-expressing cells. **c**, Method scalability comparison on 3.94 million cells. PreTSA completes in 25 s with 64.7 GB; SPARK-X fails due to R’s 32-bit integer limit; scBSP requires 108 GB memory. **d**, Number of significant genes detected by each method at p<0.05 (raw) and q<0.05 (FDR-corrected). FLASHS and PreTSA detect all 550 panel genes; scBSP detects only 1 gene after FDR correction. **e**, Robustness of SVG rankings to technical covariate adjustment. Bars show Kendall τ (versus unadjusted baseline) and top-*k* Jaccard similarity (k=50,100,200) after adjusting for log library size (C1) and section fixed effects with local cell density (C2). Moderate concordance after adjustment indicates that many spatial signals persist after controlling for technical covariates, while a subset of genes is re-ranked (Supplementary Fig. 5). **f**, Rankings of 12 known neurotransmitter system markers across methods (PreTSA ranked by F-statistic to break p-value ties). Lower rank indicates stronger spatial signal.

To demonstrate scalability on real data, we applied FLASHS and four competing methods to the Allen Brain Cell Atlas MERFISH dataset[25], which profiles 550 targeted genes across 3,938,808 cells spanning the entire adult mouse brain (Fig. 6). This dataset represents one of the largest spatial transcriptomics experiments to date and provides a stringent test of computational scalability.

FLASHS completed SVG detection across all 550 genes in 12.6 minutes with a peak memory footprint of 21.5 GB, identifying all 550 genes as spatially variable ($p<10^{-15},\;q<0.05$). This is biologically expected: the MERFISH panel comprises cell-type marker genes selected for maximal discriminative power across ~5,000 transcriptomic clusters[25], and because brain cell types are spatially organized, these markers inherently exhibit spatial expression patterns. A well-powered test on nearly four million cells should detect even subtle spatial structure.

Spatial expression maps of representative markers confirm the expected biology: *Slc17a7* shows strong cortical and hippocampal enrichment characteristic of glutamatergic projection neurons, *Gad2* marks distributed GABAergic interneurons, *Th* exhibits the highly focal midbrain expression expected of dopaminergic neurons, and *Gfap* reveals the widespread astrocytic distribution (Fig. 6a).

Of the four comparison methods, PreTSA completed in 25 seconds with 64.7 GB memory and detected all 550 genes as significant, though its marker gene rankings differed substantially from FLASHS (Kendall τ=0.27; Fig. 6f). SPARK-X failed entirely due to R’s 32-bit integer indexing limit at this scale (Fig. 6c). scBSP completed but required 108 GB of peak memory—five times that of FLASHS—and detected only a single gene after FDR correction, suggesting limited sensitivity or overly conservative inference at this scale (Fig. 6d). Moran’s I (Squidpy) completed with 35.9 GB memory and detected 543 of 550 genes at p<0.05, though it provides no multi-scale inference.

To validate biological relevance, we examined the rankings of 12 established neurotransmitter system markers spanning 8 cell populations (glutamatergic, GABAergic, dopaminergic, serotonergic, cholinergic, astrocytic, endothelial, and interneuron; see Methods for gene list and Fig. 6f). All 12 markers were detected as highly significant by FLASHS ($p<10^{-15}$), with *Slc17a7* ranked first. PreTSA’s memory (64.7 GB) already exceeds standard workstation limits on this 550-gene panel, and scales as O(n⋅g), making whole-transcriptome analysis infeasible at this cell count (see Computational scalability). FLASHS additionally provides finer-grained gene rankings: although p-values saturate near machine precision for both methods ($p<10^{-15}$; only 23 unique p-values for PreTSA), FLASHS’s spatial effect size—the ratio of observed to expected test statistic under the null—yields 550 unique ranks, whereas PreTSA’s p-values resolve only 23 distinct values; ranking PreTSA by its underlying F-statistic recovers full resolution, and Fig. 6f uses this fairer F-statistic ranking.

To validate that detected spatial patterns reflect genuine biology rather than technical artifacts, we performed covariate adjustment on the MERFISH data, regressing out log library size (C1) and section fixed effects with local cell density (C2) via Frisch–Waugh–Lovell demeaning prior to spatial testing. Technical adjustment preserved the overall scale of the effect sizes (median attenuation ratio 1.11; Fig. 6e) but produced moderate reordering of gene ranks (C1: τ=0.56; C2: τ=0.45), indicating that many high-signal genes remain robust after adjustment while a subset is meaningfully reranked. As a complementary validation, we ran FLASHS independently within individual brain sections (mean τ=0.53) and within single cell types (mean τ=0.41; Supplementary Fig. 5), supporting the view that spatial patterns persist within homogeneous subpopulations and are not reducible to library size or tissue-level composition alone.

## Discussion

The central advance of this work is resolving a long-standing trade-off between kernel expressiveness and computational scalability for spatial gene testing. The mechanism is representational: sampling hundreds of stochastic frequencies from a Gaussian kernel’s spectral density creates a richer spatial basis than the fixed, low-rank projections of existing scalable methods. Each of SPARK-X’s periodic cosine transforms probes a single spatial frequency, whereas Gaussian RFF samples from the full spectral density of a shift-invariant kernel, which by Bochner’s theorem can approximate any stationary dependence structure; furthermore, SPARK-X lacks binary and rank channels for zero-inflation robustness and omits the kurtosis correction that becomes essential at high sparsity. PreTSA’s B-spline basis spans a fixed polynomial space whose resolution is determined by knot placement. Both approaches achieve tractability by constraining the class of detectable patterns, and the benchmark and real-tissue analyses confirm that these constraints translate to measurable detection differences (Figs. 3d, 5e,f). A distinct spectral route enters spatial transcriptomics through graph signal processing: SpaGFT[26] applies Laplacian eigendecomposition on a *k*-nearest-neighbor graph, providing a topology-adaptive representation suited to multi-task analysis including SVG detection. On the SVG detection benchmark, however, the two spectral approaches yield markedly different accuracy (Fig. 2a), reflecting the distinction between discrete graph eigenbases and continuous kernel spectral sampling with universal approximation guarantees.

The practical consequence is that SVG ranking—not just significance—shapes downstream biological interpretation. Across two tissues and two methodologically distinct comparators, a consistent asymmetry emerges: FLASHS-unique SVGs converge on tissue-appropriate pathways while competitor-unique detections lack coherent enrichment. That this pattern extends to genome-wide module completeness and replicates in an independent cohort (Fig. 5) argues that the detection gap reflects genuine spatial biology captured by the multi-scale kernel but missed by lower-dimensional spatial representations. Cross-platform concordance across Visium, Slide-seq V2, and MERFISH further supports this view: spatial signals detected by FLASHS replicate across technologies despite order-of-magnitude differences in cell count and fundamentally different measurement modalities (Fig. 4d–f). For practitioners, the implication is concrete: the choice of spatial model can determine which functional programs are prioritized for downstream validation.

Three design choices complement the kernel representation and prioritize robustness across diverse data characteristics. Operating on raw counts preserves spatial signal that library-size normalization can attenuate when sequencing depth covaries with tissue position. The three-part test acts as a minimax safeguard: no single channel dominates on average, but each prevents worst-case failures on datasets where the others underperform (Supplementary Fig. 1e–i). The Cauchy combination across bandwidth scales ensures that the most informative spatial frequency drives the final p-value without dilution by uninformative scales. Together, the binary and rank channels also confer intrinsic robustness to library-size confounding, as confirmed by the covariate adjustment analysis on the MERFISH atlas (Supplementary Fig. 5c,d).

At atlas scale, memory efficiency becomes the binding constraint. FLASHS’s pergene working memory of O(D)—independent of cell count—enables million-cell analysis on standard workstations, whereas methods with O(n⋅g) memory scaling become impractical beyond ~200,000 cells without high-memory infrastructure. When p-values saturate at machine precision, as occurs for all methods at extreme sample sizes, a continuous effect-size metric preserves meaningful gene ordering for biological prioritization. We note that PreTSA achieves faster wall-clock time at all tested scales through shared dense matrix operations; FLASHS’s primary advantage lies in detection accuracy and memory efficiency, and on typical Visium-scale datasets it completes in 1–2 minutes, adequate for routine analysis.

Several limitations warrant discussion. The Open Problems benchmark generates spatial patterns via Matérn Gaussian process smooths; although multiple lines of evidence argue against systematic bias toward kernel detectors—classical GP methods perform poorly (τ<0.68), FLASHS uses a different kernel family, and the alpha-mixing metric is model-agnostic—benchmarks incorporating diverse generative models would further strengthen evaluation. The kurtosis-corrected Satterthwaite approximation brings false positive rates close to nominal across sparsity levels, but two-parameter moment matching cannot fully capture higher-order tail behavior of zero-inflated projections; saddlepoint approximations could tighten calibration at increased computational cost. The Gaussian kernel is stationary, precluding spatially adaptive detection resolution, though the multi-scale bandwidth ensemble partially mitigates this. The method has been validated on two-dimensional data; performance on three-dimensional tissue architectures from emerging volumetric technologies remains to be characterized. Finally, FLASHS identifies globally spatially variable genes but does not perform spatial domain detection or cell-type-specific spatial testing, which are complementary analytical tasks.

The frequency-domain framework opens natural extensions. Non-Gaussian spectral densities such as spectral mixture kernels[27] could improve detection of oscillatory patterns—the RFF sampling mechanism makes kernel replacement straightforward, though adapting mixture hyperparameters to the moment-based null distribution remains an open problem. Incorporating spatial covariates would enable conditional SVG detection, and generalization to multivariate statistics could identify co-varying gene programs beyond univariate spatial variability. As spatial atlases scale to whole organs and organisms, the principle that spectral transformations unlock sparsity-aware kernel computation may find application across the broader landscape of high-throughput spatial biology.

## Methods

### Random Fourier Feature representation of spatial coordinates

To test for spatial variation without constructing n×n distance matrices, FLASHS employs Random Fourier Features (RFF)[9]. By Bochner’s theorem, shift-invariant positive definite kernels can be represented as Fourier transforms of probability measures (Supplementary Note 1).

For each spatial location $s_{i}\in R^{2}$, we construct a D-dimensional feature vector:

(1) $$z\left(s_{i}\right)=\sqrt{\frac{2}{D}}{\left[cos\left(\omega_{1}^{⊤}s_{i}+b_{1}\right),\ldots ,cos\left(\omega_{D}^{⊤}s_{i}+b_{D}\right)\right]}^{⊤}$$

where $\omega_{k}\sim 𝒩\left(0,\sigma^{-2}I\right)$ are frequency vectors sampled from the spectral density of a Gaussian kernel with bandwidth σ, and $b_{k}\sim Uniform(0,\;2\pi )$ are phase offsets. The inner product $\left\langle z(s),z\left(s^{\prime }\right)\right\rangle$ approximates the Gaussian kernel $k\left(s,s^{\prime }\right)=exp(-{\left\|s-s^{\prime }\right\|}^{2}/2\sigma^{2})$.

### Adaptive multi-scale bandwidth selection

Spatial expression patterns in tissue span multiple characteristic scales, from cellular neighborhoods to tissue-wide gradients. Rather than using fixed quantile-based bandwidths, FLASHS employs adaptive bandwidth selection based on intrinsic geometric properties of the spatial point cloud. The local scale is set by the median nearest-neighbor distance, capturing fine-grained structure. The global scale is estimated from the Fiedler value[28] (second smallest eigenvalue of the graph Laplacian), providing the characteristic length scale $\ell_{\text{spectral}}=1/\sqrt{\lambda_{2}}$ for tissue-wide gradients.

FLASHS generates L (default 7) bandwidth values log-spaced between local and global scales. The D random features are distributed across scales via divmod(D,L): each scale receives ⌊D/L⌋ features, with the first D mod L scales receiving one extra.

### SVG detection via sparse sketching

For each gene, FLASHS tests whether standardized expression y associates with spatial features Z. The test statistic is:

(2) $$T={\left\|y^{⊤}Z\right\|}_{2}^{2}=\sum_{k=1}^{D}{\left(\sum_{i=1}^{n}\;y_{i}z_{ik}\right)}^{2}$$

The key computational strategy is computing this statistic without materializing the n×D feature matrix Z, leveraging sparse sketching[29] (Supplementary Note 2). For sparse expression vectors with nnz non-zero entries:

(3) $$v_{k}=\sqrt{\frac{2}{D}}\sum_{i:y_{i}\neq 0}y_{i}cos\left(\omega_{k}^{⊤}s_{i}+b_{k}\right)$$

achieving O(nnz⋅D) per gene. Centering is performed implicitly by pre-computing the column sums $1^{⊤}Z$ once during model fitting and subtracting $\overset{‾}{y}\left(1^{⊤}Z\right)$ from the sparse projection, preserving sparsity (Supplementary Note 2). The centering vector $1^{⊤}Z$ is computed exactly in one O(nD) pass (with O(D) memory) rather than subsampled. This is required for calibration: centering error enters the quadratic statistic as ${\left\|\overset{‾}{y}\delta \left(1^{⊤}Z\right)\right\|}^{2}$, which can dominate the null scale when n is much larger than the subsample size used for moment estimation.

### Three-part test for zero-inflated spatial data

To handle zero-inflation, FLASHS applies three complementary test channels per gene and per bandwidth scale:

1. **Binary test**: Tests whether the indicator 1(y>0) shows spatial structure, capturing patterns in gene detection.
2. **Rank test**: Tests whether rank-transformed non-zero values vary spatially, providing outlier-robust intensity assessment.
3. **Direct test**: Tests whether raw expression values show spatial structure, preserving library-size-correlated signal.

In addition to the multi-scale RFF kernels, FLASHS includes a linear spatial trend test for each channel, regressing the (binary, rank, or direct) expression vector onto centered spatial coordinates. This test detects tissue-wide linear gradients that may fall between Gaussian bandwidth scales. The three resulting p-values ($\chi_{d}^{2}$ under the null, d = spatial dimensionality) enter the Cauchy combination alongside the RFF-based p-values.

### Cauchy combination across scales and test types

Per-scale p-values from all three test types are combined via the Cauchy combination test[10]:

(4) $$T_{CCT}=\frac{1}{K}\sum_{j=1}^{K}tan\left[\left(\frac{1}{2}-p_{j}\right)\pi \right]$$

where K=3L+3: three test channels across L bandwidth scales, plus three linear spatial trend tests. Under the null, $T_{CCT}$ follows a standard Cauchy distribution regardless of the dependency structure among the K component tests, requiring only that each marginal p-value is valid under the null; this yields a single combined p-value per gene.

### Null distribution and p-value computation

Under the null hypothesis of no spatial variation, the test statistic $T=\sum_{k}v_{k}^{2}$ where $v_{k}=y^{⊤}z_{k}$ (centered). Since the RFF columns $z_{k}$ are correlated—particularly at large bandwidth scales where multiple cosine features approximate similar low-frequency polynomials—the standard Satterthwaite approximation that assumes independent $v_{k}$ underestimates the null variance. Specifically, for jointly normal $v_{k}$, $Var[T]=2{\left\|\Sigma_{v}\right\|}_{F}^{2}$ where $\Sigma_{v}=\sigma_{y}^{2}Z_{c}^{⊤}Z_{c}/n$ is the covariance of the projection vector, and $Z_{c}$ denotes the column-centered feature matrix.

We use a kurtosis-corrected Satterthwaite moment matching[30] with $E\left[T_{\ell }\right]=n\sigma_{y}^{2}\sum_{k}Var\left[z_{k,\ell }\right]$ and the per-scale variance:

$$Var\left[T_{\ell }\right]=2\sigma_{y}^{4}n^{2}{\left\|\Sigma_{z,\ell }\right\|}_{F}^{2}+\kappa_{4}\sigma_{y}^{4}\sum_{i=1}^{n}{\left\|z_{c,\ell ,i}\right\|}^{4}$$

where $\Sigma_{z,\ell }$ is the centered feature covariance within scale ℓ and $\kappa_{4}=E[{\left(\tilde{y}/\sigma_{y}\right)}^{4}]-3$ is the per-gene excess kurtosis[31]. The second term—a kurtosis correction not present in standard quadratic-form approximations used by existing SVG methods—accounts for the leptokurtic distribution of zero-inflated expression vectors ($\kappa_{4}\gg 0$), which causes the Gaussian-only formula to underestimate the null variance and inflate p-values (Supplementary Note 3). Both terms are estimated once on a coordinate subsample (M=10,000 by default) at $O\left(M\sum_{\ell =1}^{L}\;D_{\ell }^{2}\right)$ cost. The scale and degrees of freedom of the approximating $\kappa \chi_{\nu }^{2}$ distribution are then $\kappa =Var\left[T_{\ell }\right]/\left(2E\left[T_{\ell }\right]\right)$ and $\nu =2E{\left[T_{\ell }\right]}^{2}/Var\left[T_{\ell }\right]$. This provides analytic p-values without permutation (see Results for FPR and FDR assessment).

### Multiple testing correction

P-values across genes are adjusted using the Benjamini–Hochberg procedure to control the false discovery rate at level α (default 0.05). This ensures a unified correction framework across all methods in comparative analyses.

### Benchmarking

We evaluated FLASHS on the Open Problems benchmark for SVG detection[11], which includes 11 published SVG detection methods on its leaderboard (see Results for dataset description). All leaderboard methods were scored with default parameters as specified in the benchmark.

Each method produces a per-gene spatial variability score; the benchmark evaluates methods by Kendall τ between predicted and ground truth scores. As in the Open Problems framework, each method uses the score most appropriate to its statistical model: SpatialDE, SpatialDE2, and SOMDE report the fraction of spatial variance (FSV); Moran’s I reports the I statistic; SpaGFT, Spanve, and Sepal report method-specific test statistics; scGCO and SpaGCN use $-{log}_{10}(p)$. FLASHS uses the spatial effect size, defined as the maximum ratio of observed to expected test statistic across the three test channels (binary, rank, direct); an ablation across all 50 datasets confirms that $-{log}_{10}(p)$ alone yields substantially lower concordance (τ=0.868), as p-values saturate at moderate sample sizes, whereas the effect size provides a continuous, non-saturating ranking metric (Supplementary Table 13).

FLASHS was applied to raw counts without library-size normalization or log-transformation, using D=500 random features, L=7 bandwidth scales, and the three-part test (binary, rank, direct). Three additional methods not included in the original leaderboard—PreTSA[8], Hotspot[7], and scBSP[6]—were benchmarked on the same 50 datasets using their default parameters. PreTSA was run using its Python implementation with default knot setting (knot = 0, corresponding to a cubic B-spline basis without internal knots) on library-size-normalized and log-transformed data as specified by the authors; PreTSA also provides an automatic knot selection mode, but we used the default configuration as recommended in the package documentation. All benchmarks were performed on compute nodes of a shared HPC cluster (AMD EPYC, 16 cores). FLASHS version 0.1.0 was used with Python 3.11, NumPy 1.26, SciPy 1.12, and Numba 0.59.

### Scalability experiments

Scalability was evaluated on simulated datasets with cell counts of 1,000, 5,000, 10,000, 25,000, 50,000, 100,000, 200,000, 500,000, and 1,000,000. Spatial coordinates were sampled uniformly in two dimensions. For each cell count, 5,000 genes were simulated from a negative binomial distribution with size parameter 1.0 and probability 0.1, yielding realistic sparsity (~90% zeros), constructed in 50,000-cell chunks to limit memory during data generation. Three independent replicates were run per condition. Runtime was measured as wall-clock time from model fitting through p-value computation, and peak memory was recorded as peak resident set size via the resource module.

Runtime comparison against nine SVG detection methods—Moran’s I (Squidpy[13]), SpatialDE[1], Hotspot[7], SPARK-X[5], SOMDE[14], scBSP[6], Spanve[15], nnSVG[4], and PreTSA[8]—was performed on the same simulated data at cell counts of 1,000, 5,000, 10,000, 25,000, and 50,000 (3 replicates each). SPARK-X and nnSVG were called via rpy2 from their R implementations. Each method–size–replicate combination was run as an isolated subprocess with a 2-hour timeout and 50 GB memory limit. Extended comparison between FLASHS and PreTSA was performed at 100,000, 200,000, 500,000, and 1,000,000 cells (Supplementary Table 4) to characterize large-scale memory and runtime scaling.

All scalability and comparison experiments were performed on compute nodes of a shared HPC cluster (AMD EPYC, 16 cores, 1 TB RAM).

### Atlas-scale MERFISH analysis

We applied FLASHS, PreTSA, SPARK-X, scBSP, and Moran’s I (Squidpy) to the Allen Brain Cell Atlas MERFISH dataset[25] (specimen C57BL6J-638850). The dataset contains 4,334,174 cells profiled for 550 targeted genes across the whole adult mouse brain. Spatial coordinates (x,y) were obtained from the published cell metadata; after matching cell identifiers between the expression matrix (H5AD format) and metadata, 3,938,808 cells with both expression and coordinate data were retained. Expression data were used as raw counts without normalization or log-transformation. Methods were evaluated on three axes: completability at this scale (whether the method terminates without error or memory exhaustion), computational resource usage (runtime and peak memory), and statistical ranking resolution (number of distinct gene ranks, relevant when most p-values saturate at machine precision).

FLASHS was run with default parameters (D=500, L=7, minimum 5 expressing cells). SPARK-X was called via rpy2 from its R implementation (SPARK package). scBSP (v0.3.1) was called via its Python interface (granp function). Moran’s I was computed via Squidpy using a *k*-nearest-neighbor graph (k=6). PreTSA was run using its Python implementation with default parameters on library-size-normalized and log-transformed data; because PreTSA p-values saturate at machine zero for most genes at this sample size, gene rankings were derived from the F-statistic to provide finer discrimination. Each method was run as an isolated SLURM job on a 16-core compute node with 128 GB memory allocation. Runtime and peak resident set size were recorded.

Biological validation used 12 established neurotransmitter system marker genes with known spatial expression patterns in the mouse brain: *Slc17a7* and *Slc17a6* (glutamatergic), *Slc32a1* and *Gad2* (GABAergic), *Slc6a3* and *Th* (dopaminergic), *Slc6a4* (serotonergic), *Chat* (cholinergic), *Aqp4* and *Gfap* (astrocytic), *Cldn5* (endothelial), and *Calb2* (interneuron).

### Structured null testing on multi-section atlas data

To characterize rejection behavior under structured null hypotheses that preserve part of the spatial organization, we applied three permutation strategies to the full Allen MERFISH atlas (3,938,808 cells, 550 genes) following the exchangeability-block framework[17]: (i) global row permutation (N0), which shuffles expression vectors across all cells, destroying all spatial structure; (ii) within-section permutation (N1), which shuffles expression only among cells within the same brain section, preserving between-section spatial structure; and (iii) spatial block permutation (N2), which partitions each section into a 6 × 6 grid and permutes expression vectors between grid blocks, preserving within-block spatial autocorrelation. For N0, 3 independent replicates of 550 genes were run (1,650 null tests); for N1, 10 replicates (5,500 tests); for N2, 6 replicates (3,300 tests). The rejection rate was computed as the fraction of genes with p<0.05; for N0, where all spatial structure is destroyed, this corresponds to the empirical false positive rate (Supplementary Table 9).

### Confounding sensitivity analysis

To test whether SVG rankings on the MERFISH atlas are driven by technical or compositional confounders, we applied FLASHS to the same 3,938,808 cells after progressively regressing out potential confounders. Covariate sets were defined as: C0 (no adjustment; baseline), C1 (technical: log total counts; no mitochondrial genes are present in the 550-gene MERFISH panel), and C2 (C1 + brain-section fixed effects + local cell density). Cell density was computed per section via 2D histogram binning (50 × 50 grid). Residualization used Frisch–Waugh–Lovell demeaning for the categorical fixed effect (section), followed by ordinary least squares projection for continuous covariates (log total counts, cell density). FLASHS was then applied to the residualized expression matrix with min_expressed = 0 (since residuals are no longer sparse). Stability was assessed by Kendall τ and top-*k* Jaccard between each adjusted ranking and the C0 baseline. Stratified analyses ran FLASHS independently within the 5 largest brain sections and 5 most abundant cell-type classes, comparing within-stratum rankings to the C0 baseline.

### Simulation studies

For Type I error assessment, null datasets were generated on a 50×50 grid (2,500 spots) at three zero-fraction levels: ~50% (Poisson, λ=0.7), ~90% (negative binomial, n=1,p=0.9), and ~95% (negative binomial with additional 50% dropout). Expression was drawn without spatial structure. Twenty replicates of 1,000 genes each were generated per sparsity level, yielding 60,000 null tests. The false positive rate was computed as the fraction of genes with p<0.05.

For power evaluation, datasets were generated on a 50 × 50 grid with 500 spatially variable genes and 500 null genes per replicate (10 replicates per condition). Three spatial pattern types were tested: (i) hotspot—localized expression with a Gaussian spatial envelope of radius 10 grid units, (ii) gradient—a linear expression gradient across the tissue, and (iii) periodic—sinusoidal expression with spatial frequency matching 3 cycles across the grid. Effect sizes (scaling factor of spatial component relative to background) were 0.1, 0.3, and 0.5. Sparsity levels (fraction of zeros added beyond natural zeros) were 10%, 30%, and 50%. True positive rate and false discovery rate were computed at q<0.05.

For the cross-method detection power comparison (Fig. 3d–f), datasets were generated on a 50 × 50 grid (2,500 spots) with 200 spatially variable and 200 null genes per replicate (10 replicates). Five spatial pattern types were tested: (i) gradient—a linear expression ramp at a random orientation across the tissue, (ii) hotspot—focal expression with a Gaussian spatial envelope centered at a random location (radius 5–15% of tissue diameter), (iii) domain boundary—a sharp step function between two expression regions separated by a line at random angle and offset, (iv) multiscale—a superposition of gradient and hotspot components (equal weight 0.5 each), and (v) periodic—a sinusoidal signal with random orientation and frequency of ~3 cycles across the tissue. Background expression was drawn from a negative binomial distribution. Effect sizes were 0.3, 0.5, and 1.0; sparsity levels were 50%, 80%, and 95% zeros. FLASHS, SPARK-X, and PreTSA were each applied with default parameters. True positive rate was computed at q<0.05 after Benjamini–Hochberg correction.

Ablation experiments varied D∈{50,100,200,500,1,000} (with L=7 fixed) and L∈{1,3,5,7,10} (with D=500 fixed), testing 200 spatially variable and 200 null genes per replicate (5 replicates per condition).

### DLPFC cross-dataset validation

We applied FLASHS and SPARK-X to 12 Visium samples from the Human DLPFC dataset[18] (3 donors, 4 samples each; 33,538 genes per sample). FLASHS was run with default parameters on raw counts. SPARK-X was run independently on all 12 samples via rpy2 for cross-method comparison. Cross-sample consistency was assessed by pairwise Kendall τ correlation of gene effect-size rankings. Marker enrichment was evaluated against 27 known cortical layer markers curated from Maynard et al. (spanning layers L1–L6 and white matter). Fold enrichment at top-*k* was computed as the fraction of markers in the top k genes divided by the expected fraction under random ranking.

### Cross-platform SVG validation

To assess whether SVG rankings generalize across measurement technologies, we compared FLASHS and PreTSA results on two independently profiled mouse brain datasets: 10x Visium V1_Adult_Mouse_Brain (2,702 spots, 32,285 genes) and the Allen MERFISH atlas (3,938,808 cells, 550 genes). For each method, gene-level rankings were computed independently on each platform. The 500 genes present in both platforms formed the common evaluation set. Cross-platform concordance was assessed by Kendall τ and Spearman ρ correlation between platform-specific rankings restricted to these common genes, with significance evaluated via 1,000-replicate permutation test (random shuffling of gene labels). Top-*k* Jaccard similarity was computed at k∈{50,100,200}. FLASHS was run with default parameters; PreTSA was run using its Python implementation (spatialTest) with default parameters on library-size-normalized and log-transformed data. MERFISH method results were loaded from cached outputs of the atlas-scale analysis.

To extend cross-platform validation to a third technology, we applied FLASHS to a Slide-seq V2 hippocampus dataset (41,786 beads, 4,000 highly variable genes) with default parameters. Gene-level spatial effect sizes were used for ranking. Pairwise concordance with existing Visium and MERFISH rankings was assessed by Kendall τ, Spearman ρ, top-*k* Jaccard (k=50,100,200), and permutation tests (1,000 replicates) on the intersection of genes present in both platforms (Slide-seq ∩ Visium: 3,666 genes; Slide-seq ∩ MERFISH: 319 genes; three-way intersection: 319 genes). Brain region marker enrichment was evaluated across all three platforms using 15 established markers spanning 7 cell populations.

### Gene Ontology enrichment analysis

To assess the biological relevance of method-specific SVG detections, we performed Gene Ontology (GO) enrichment analysis on the Visium human heart dataset (10x Genomics, 4,235 spots). FLASHS was applied to 16,716 genes passing its expression filter (at least 5 expressing cells); restricting to the 14,634 genes tested by both FLASHS and PreTSA, genes were categorized as detected by both methods, FLASHS-only, PreTSA-only, or neither, based on significance at q<0.05 after uniform Benjamini–Hochberg correction. GO enrichment was performed using g:Profiler[32] with all 14,634 commonly tested genes as background, querying GO Biological Process, GO Molecular Function, GO Cellular Component, KEGG, and Reactome databases. Statistical significance was assessed using the g:SCS method with a threshold of p<0.05.

For the DLPFC cross-dataset analysis, FLASHS and SPARK-X were compared on 12 Visium samples (3 donors, 4 samples each; 33,538 genes). For each sample, genes tested by both methods formed the common gene universe. Both methods’ raw p-values were corrected via Benjamini–Hochberg within each sample’s common gene universe. Genes were categorized as FLASHS-unique, SPARK-X-unique, both, or neither at q<0.05. GO enrichment of method-specific gene sets was performed via the g:Profiler REST API with the per-sample common gene universe as custom background, using the same source databases and significance threshold as above.

### Heart metabolic pathway enrichment and deconvolution validation

To investigate the biological program underlying the FLASHS-unique SVGs in the human heart, we performed hypergeometric enrichment analysis against curated gene sets from the Molecular Signatures Database (MSigDB)[19]. Five gene sets were tested: PPARGC1A_TARGET_GENES (78 of 83 PGC-1*α* transcriptional targets present in the 14,634-gene universe), HALLMARK_OXIDATIVE_PHOSPHORYLATION (199 genes), a curated mitochondrial biogenesis set (49 genes spanning mitochondrial ribosomal proteins, translocase complexes, and respiratory chain assembly factors), KEGG_DILATED_CARDIOMYOPATHY (71 of 90 pathway genes in universe), and HALLMARK_MYOGENESIS (177 of 200 hallmark genes in universe). Enrichment was computed as one-sided hypergeometric tests with the 14,634 tested genes as background. Genes were stratified into four categories based on FLASHS and PreTSA detection at q<0.05 (Benjamini–Hochberg correction for both methods): shared (both methods), FLASHS-only, PreTSA-only, and neither.

For spatial deconvolution, we used the Litviňuková et al. human heart single-cell RNA-seq atlas[22] (486,134 cells; 452,136 retained after removing unassigned/doublet categories across 12 cell types). Deconvolution was performed using FlashDeconv[23] with default parameters (sketch dimension 512, automatic spatial regularization, 2,000 HVGs, log-CPM preprocessing). Per-spot cell-type proportions were estimated for 4,235 Visium spots. A mitochondrial SVG module score was computed per spot as the mean log_1p_-normalized expression (CPM-scaled) of the 49 curated mitochondrial biogenesis genes. Spearman rank correlation was computed between the module score and each deconvolved cell-type proportion.

To compare detection sensitivity of the 49 mitochondrial biogenesis genes across non-parametric methods, Moran’s I was computed on the same Visium heart dataset via Squidpy[13] (k=6 nearest neighbors) on all 36,601 genes, and SPARK-X was run via rpy2 with default parameters on the 14,634 filtered genes. P-values for all methods were corrected using Benjamini–Hochberg at q<0.05.

To account for spatial autocorrelation in the correlation test, we performed spatial block permutation testing. The tissue was divided into a regular grid (m×m, tested at m∈{6,8,10}), and block labels were permuted 10,000 times while preserving within-block spatial structure. Two-sided permutation p-values were computed as the fraction of permutations where $\left|\rho_{\text{perm}}\right|\geq \left|\rho_{\text{obs}}\right|$. To test for gene-set leakage, we re-ran FlashDeconv after removing all 49 mitochondrial biogenesis genes from the Visium expression matrix, then recomputed the correlation between the original module score and the leakage-free deconvolved cell-type proportions.

To assess whether the detection advantage extends beyond individual pathways, we computed module completeness across 365 curated gene sets (50 MSigDB Hallmark 2020 and 315 KEGG 2021 Human pathways, downloaded via the Enrichr REST API). For each gene set G and method M, module completeness is defined as $|G\cap {SVG}_{M}\cap U\left|/|G\cap U|\right.$, where U is the set of 14,634 tested genes and ${SVG}_{M}$ is the set of genes significant at q<0.05 for method M. Gene sets with fewer than 5 genes in U were excluded. All methods use Benjamini–Hochberg correction; for Moran’s I, genome-wide BH-corrected SVGs (computed via Squidpy 1.6.1, k=6 nearest neighbors, on 36,601 genes) were intersected with U.

### External replication on independent cardiac cohort

To assess the reproducibility of the mito–cardiomyocyte association, we replicated the full analysis on four healthy control Visium slides from Kuppe et al.[24] (patients P1, P7, P8, P17; 2,043–4,269 spots per slide, 13,144–15,730 genes after filtering). Raw counts were extracted from the adata.raw layer of each h5ad file; spatial coordinates were taken from obsm[‘X_spatial’]. FLASHS and PreTSA were run with the same parameters as the 10x Heart analysis. Gene categorization (shared/FLASHSonly/PreTSA-only/neither) and hypergeometric enrichment testing used the same pipeline and gene sets. Pre-computed cell2location[33] deconvolution proportions provided in the Kuppe h5ad files were used directly for cell-type correlation analysis. Because all four control slides are from the left ventricle, the “Cardiomyocyte” proportion column serves as a ventricular cardiomyocyte proxy. The transferred 49-gene mito module score and spatial block permutation tests (m∈{6,8,10}, 10,000 permutations) followed the same protocol as the discovery analysis. Only 16 of 49 mitochondrial biogenesis genes were present in the Kuppe Visium gene panel.

### Implementation

FLASHS is implemented in Python using NumPy and SciPy for linear algebra and Numba[34] for JIT-compiled parallel kernels. Key optimizations include sparse sketching for O(nnz⋅D) projection (with full per-gene complexity O(nnz⋅D+nnz⋅lognnz) including rank transformation), implicit centering to preserve sparsity, and a fused triple projection kernel that evaluates all three test types in a single pass over the non-zero entries. Default parameters: D=500 random features, L=7 bandwidth scales. Source code is available at https://github.com/cafferychen777/FlashS. Model fitting uses one exact O(nD) centering pass plus subsampled moment estimation for variance terms; this keeps inference calibrated while preserving scalability in the gene-wise testing stage.

## Supplementary Material

Supplement 1

Supplementary information is available for this paper.

## Data Availability

The Allen Brain Cell Atlas MERFISH data are publicly available at https://portal.brain-map.org/atlases-and-data/bkp/abc-atlas. The Visium human heart dataset is publicly available from 10x Genomics (https://www.10xgenomics.com/datasets/human-heart). The Human DLPFC Visium data[18] are available via the spatialLIBD Bioconductor package (http://spatial.libd.org/spatialLIBD/). The Slide-seq V2 hippocampus dataset[35] is available from the Broad Institute Single Cell Portal. Open Problems benchmark datasets are available through the Open Problems framework[11]. The Kuppe et al. healthy control Visium slides[24] are available from Zenodo (https://zenodo.org/records/6578047).

## Code availability:

FLASHS is freely available at https://github.com/cafferychen777/FlashS under the MIT license.
