# Multi-scale spatial testing recovers gene programs missed by existing detection methods

**DOI:** 10.64898/2026.03.12.711372

**Published:** 2026-04-09

**Authors:** Chen Yang, Xianyang Zhang, Jun Chen

**Affiliations:** 1 Department of Statistics, Texas A&M University, College Station, Texas, 77843, USA; 2 Division of Computational Biology, Department of Quantitative Health Sciences, Mayo Clinic, Rochester, Minnesota, 55905, USA

**Keywords:** spatially variable genes, spatial transcriptomics, random Fourier features, kernel methods, single-cell genomics

## Abstract

Identifying spatially variable genes (SVGs) is the first analytical step in spatial transcriptomics, determining which genes and pathways are prioritized for downstream validation. Yet the restricted spatial models of current detection methods create systematic blind spots that can exclude biologically coherent programs from discovery. Here we present FlashS, which reformulates kernel-based spatial testing in the frequency domain to detect arbitrary multi-scale expression patterns while scaling to millions of cells. In human cardiac tissue, this broader detection capacity recovers a coherent PGC-1*α*-regulated mitochondrial biogenesis program—40 of 49 pathway genes spatially associated with ventricular cardiomyocytes—that PreTSA, a leading parametric alternative, largely misses (1 of 49 genes), a finding replicated in an independent cohort. Across 50 benchmark datasets spanning 9 platforms, FlashS achieves state-of-the-art ranking accuracy (mean Kendall τ=0.935) and completes on the Allen Brain MERFISH atlas (3.94 million cells) in 12.6 minutes with 21.5 GB memory.

## Introduction

Spatial transcriptomics technologies now measure gene expression while preserving its tissue context, enabling systematic discovery of spatially variable genes (SVGs)—genes whose expression patterns carry spatial structure beyond random fluctuation. SVGs underlie fundamental biological processes: developmental morphogen gradients that pattern embryonic tissues, metabolic zonation across the liver lobule, immune cell infiltration at tumor margins, and regionalized transcriptional programs in the brain cortex. Identifying these genes is often the first analytical step toward understanding how spatial organization shapes tissue function.

Existing SVG detection methods span a range of statistical models and scalability profiles. Gaussian process (GP)-based approaches[1–4] provide principled likelihood-ratio tests but require $O\left(n^{2}\right)-O\left(n^{3}\right)$ covariance matrix construction. Scalable alternatives—including covariance projection (SPARK-X[5]), granularity-based testing (scBSP[6]), local autocorrelation (Moran’s I; Hotspot[7]), and B-spline regression (PreTSA[8])—achieve tractability by restricting the spatial model to low-rank projections, fixed polynomial bases, or single-scale statistics. This creates a common constraint: universal kernels (Gaussian, Matérn) that detect the broadest class of spatial patterns are computationally expensive, while scalable methods sacrifice detection capacity.

A further challenge is zero-inflation: sequencing-based platforms routinely produce data with 80–95% zero entries per gene, reflecting both biological absence and technical dropout. Most SVG detection methods operate on normalized or log-transformed data without explicitly modeling the zero component, potentially obscuring spatial patterns encoded in the presence–absence structure and attenuating genuine signal when sequencing depth covaries with tissue position. Despite substantial methodological progress, no existing method simultaneously achieves high detection accuracy, computational scalability to million-cell datasets, and robustness to the extreme zero-inflation characteristic of spatial transcriptomics data.

Our key insight is that the kernel–scalability trade-off can be resolved by reformulating spatial testing in the frequency domain. Random Fourier Features (RFF)[9] approximate kernel evaluations via inner products in a compact spectral space, making the Gaussian kernel—whose universal approximation capacity is ideal for detecting arbitrary spatial patterns—computationally tractable without constructing any n×n matrix. This reformulation is naturally suited to spatial transcriptomics: multi-scale patterns map onto distinct frequency bands and are captured simultaneously, while expression sparsity translates directly into computational savings, as per-gene cost scales with the number of expressing cells rather than total cell count.

Building on this foundation, we incorporate two additional elements for robustness. First, to handle the extreme zero-inflation of spatial transcriptomics data, a three-part test separately probes binary (presence/absence), rank (intensity order), and raw-count (absolute abundance) aspects of spatial expression, targeting complementary information that no single channel captures. Second, because spatial patterns span multiple characteristic scales—from cellular neighborhoods to tissue-wide gradients—per-scale p-values are combined via a Cauchy combination rule[10], which aggregates evidence across bandwidth scales and test types without requiring knowledge of their dependency structure.

We present FlashS (**F**requency-domain **L**arge-scale **A**nalysis of **S**patial **H**eterogeneity), a framework implementing these ideas. Across 50 benchmark datasets spanning 9 spatial transcriptomics platforms[11], FlashS achieves state-of-the-art ranking accuracy while scaling to atlas-scale datasets of nearly four million cells on standard hardware. We show that this accuracy advantage has concrete implications for biological interpretation: in human cardiac tissue, FlashS recovers a coherent mitochondrial biogenesis program associated with ventricular cardiomyocytes that is largely invisible to parametric alternatives, a finding replicated in an independent cohort.

## Results

### Overview of FlashS

**Fig. 1:**
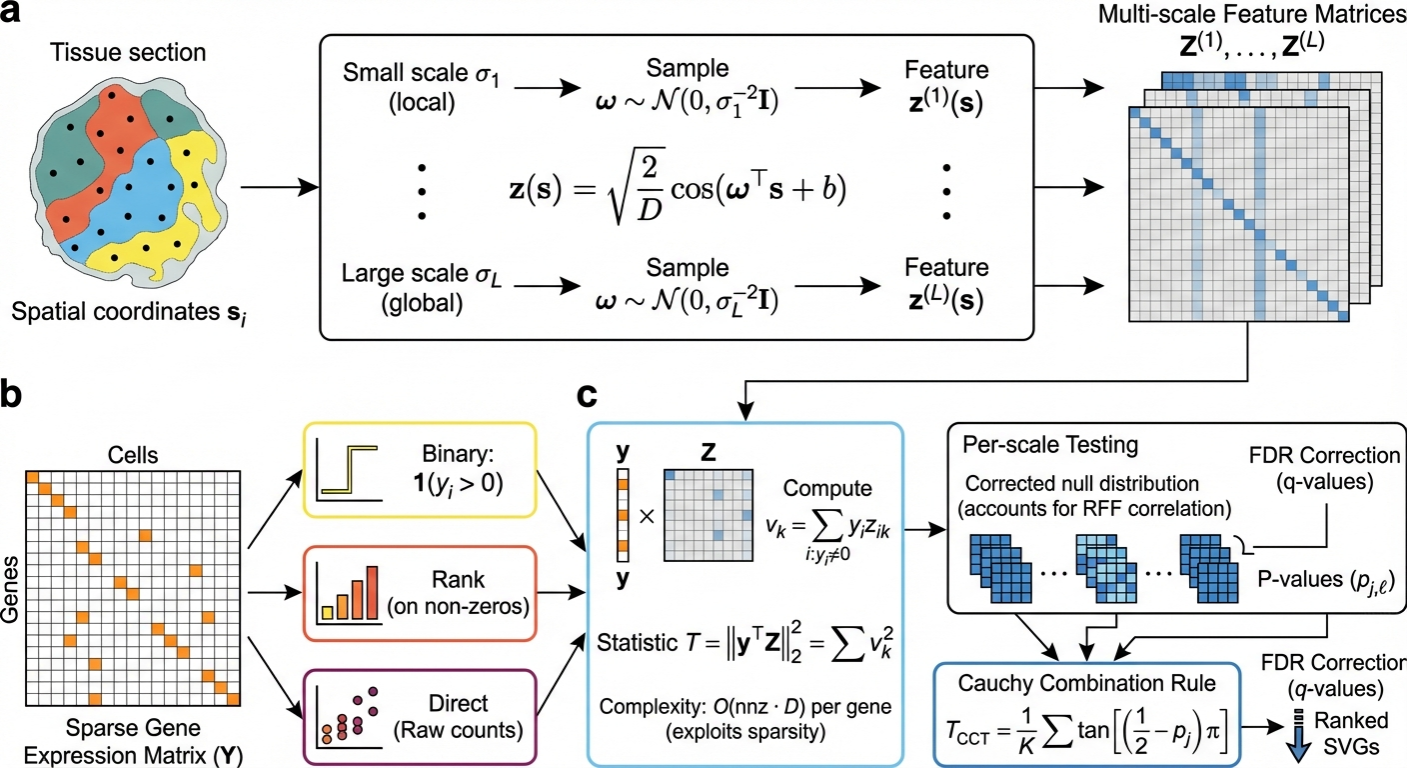
Overview of FlashS. **a**, Spatial coordinates are transformed into Random Fourier Features (RFF), approximating kernel evaluations via inner products in a low-dimensional feature space. **b**, A three-part test evaluates binary expression, rank-transformed intensities, and raw counts against spatial features at multiple bandwidth scales. **c**, The test statistic $T={\left\|Z^{⊤}y\right\|}^{2}$ aggregates squared projections of expression onto spatial features. Per-scale p-values are combined via the Cauchy combination rule to yield a single p-value per gene.

For each gene, FlashS tests whether expression depends on spatial location or is spatially independent, using Random Fourier Features (RFF) to make this test computationally tractable (Fig. 1). RFF transforms spatial coordinates into a compact feature representation whose inner products approximate Gaussian kernel evaluations, converting an $O\left(n^{2}\right)$ kernel computation into O(n⋅D) linear operations, where D is the number of random features (D=500 by default). The test statistic measures the total squared correlation between gene expression and these spatial features: genes with spatial structure produce large projections onto specific frequency components, while spatially independent genes do not. To handle the extreme zero-inflation of spatial transcriptomics, a three-part test evaluates binary expression patterns (presence/absence), rank-transformed intensities, and raw counts, capturing complementary spatial signals that no single representation would detect. Per-scale p-values from all test components are merged via a Cauchy combination rule[10] across multiple bandwidth scales, yielding a single p-value per gene.

A key design choice enables FlashS to turn expression sparsity from a nuisance into a computational advantage. Because spatial transcriptomics data are typically 80–95% zeros, the projection of expression onto spatial features needs to access only the non-zero entries. An implicit centering strategy (Methods; Supplementary Note 2) preserves this sparsity, so that per-gene cost scales with the number of expressing cells rather than total cell count—a property that becomes increasingly advantageous as datasets grow to millions of cells while per-gene sparsity remains high.

FlashS has two main tuning parameters: the number of RFF features D (default 500) and the number of bandwidth scales L (default 7). The bandwidths $\sigma_{1},\ldots ,\sigma_{L}$ are selected adaptively from data-intrinsic length scales rather than specified by the user (Methods). Since the total feature count D is fixed, changing L adjusts scale resolution without affecting per-gene cost, which remains O(nnz⋅D). Sensitivity analysis confirms that the defaults provide a robust operating point across all 50 benchmark datasets (Supplementary Fig. 1a–d). Two design choices have substantial impact on accuracy: operating on raw counts rather than library-size-normalized data improves mean τ by 0.15 across all 50 datasets (Supplementary Table 6), and the three-part test raises the worst-case per-dataset τ from 0.72 (binary channel alone) to 0.79 (full test), providing robustness against datasets where any single channel underperforms (Supplementary Fig. 1e–i).

### Benchmark accuracy on the Open Problems SVG evaluation

We evaluated FlashS on the Open Problems benchmark for SVG detection[11], a standardized evaluation framework comprising 50 datasets across 9 spatial transcriptomics platforms (Visium, Slide-seq V2, MERFISH, STARmap, Stereo-seq, seqFISH, DBiT-seq, Slide-tags, and Post-Xenium). Datasets span diverse organisms (human, mouse, *Drosophila*) and tissues (brain, cancer, embryo, heart, kidney, and others), with sizes ranging from 936 to 29,309 spots and 210 to 1,050 genes per dataset. Ground truth spatial variability scores were generated using scDesign3[12] by mixing Gaussian process and non-spatial models. Performance is measured by Kendall τ correlation between predicted and true spatial variability scores, averaged across genes grouped by their original feature identity.

**Fig. 2:**
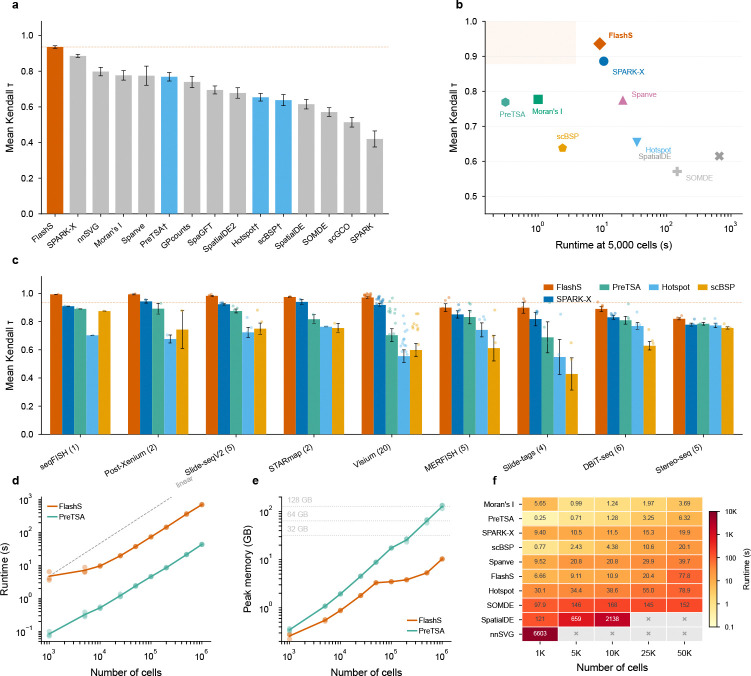
Benchmark accuracy, scalability, and per-platform performance. **a**, Mean Kendall τ correlation between predicted and ground-truth spatial variability scores across 50 datasets spanning 9 platforms. FlashS (red) achieves τ=0.935, surpassing SPARK-X (τ=0.886) by Δτ=0.049. PreTSA, Hotspot and scBSP (marked with †) were benchmarked independently on the same datasets. Error bars indicate standard error of the mean. **b**, Accuracy versus runtime (at 5,000 cells). FlashS occupies the Pareto-optimal region of high accuracy and fast runtime (shaded). **c**, Per-platform mean Kendall τ for FlashS, SPARK-X, PreTSA, Hotspot, and scBSP across 9 platforms, sorted by FlashS performance. Individual dataset values shown as dots. **d**, Runtime as a function of cell number (5,000 genes). Both FlashS and PreTSA scale near-linearly; dashed line indicates linear scaling. **e**, Peak memory usage. PreTSA’s memory scales as O(n⋅g), crossing 32 GB at ~200,000 cells and reaching 137 GB at 1,000,000 cells; FlashS remains below 11 GB via sparse sketching. Dotted lines mark typical workstation memory. Points show individual replicates (n=3 per size). **f**, Runtime heatmap for 10 SVG detection methods across cell counts (1,000–50,000), sorted by speed at 50,000 cells. Cell values show mean runtime in seconds; × marks timeout (>2 h) or out-of-memory failure. All benchmarks performed on a 16 -core compute node.

FlashS achieves a mean Kendall τ of 0.935 across all 50 datasets, establishing a new state of the art (Fig. 2; Supplementary Table 1). This surpasses the previous best method, SPARK-X (τ=0.886), by Δτ=0.049. We independently validated this comparison by running SPARK-X in the same computing environment on all 50 datasets, obtaining τ=0.881 (consistent with the leaderboard value), with FlashS outperforming on every dataset (Δτ=+0.054; Supplementary Table 10). Notably, FlashS maintains positive correlation across all 50 datasets (minimum τ=0.788), whereas several competing methods produce negative correlations on individual datasets, indicating anti-correlated predictions. Performance is consistent across all 9 platforms, with mean τ>0.80 on every platform (Supplementary Fig. 2).

To contextualize the accuracy–scalability trade-off, we additionally benchmarked three scalable methods not included in the original Open Problems leaderboard—PreTSA[8], Hotspot[7], and scBSP[6]—on the same 50 datasets (Fig. 2a). PreTSA, which applies B-spline tensor product regression with an F-test, achieves mean τ=0.769; Hotspot achieves mean τ=0.654 and scBSP mean τ=0.638. Despite its computational efficiency, PreTSA’s parametric regression captures smooth spatial trends but cannot model the multi-scale, non-smooth patterns that kernel-based methods detect, resulting in a Δτ=0.166 gap relative to FlashS. On the accuracy–runtime Pareto frontier (Fig. 2b), FlashS is more accurate than other scalable alternatives and faster than methods of comparable accuracy.

### Computational scalability

Emerging spatial atlases profile millions of cells across entire organs, demanding SVG detection methods that scale without sacrificing accuracy. We benchmarked FlashS on simulated datasets with 5,000 genes and cell counts ranging from 1,000 to 1,000,000 (Fig. 2d,e). Runtime scales near-linearly with cell number, reaching 11.7 minutes at one million cells with peak memory of 10.3 GB—within the capacity of a standard workstation (Supplementary Table 2).

This memory efficiency distinguishes FlashS from the only other method of comparable accuracy. Among ten compared methods (Fig. 2f; Supplementary Table 3), PreTSA achieves the lowest wall-clock time but its memory scales as O(n⋅g), reaching 137 GB at one million cells—13-fold more than FlashS (Supplementary Table 4). On a typical workstation (32–64 GB RAM), PreTSA becomes infeasible beyond ~200,000 cells. GP-based methods (SpatialDE, nnSVG) exceed the 2-hour timeout at ≥5,000 cells (Supplementary Table 3). Among methods with competitive accuracy (τ>0.85), FlashS is the fastest.

### Statistical calibration under the null

Accurate p-values are essential for controlling false discoveries when thousands of genes are tested simultaneously. To assess calibration, we generated null datasets on 2,500-spot grids at three zero-fraction levels (~50%, ~90%, and ~95%) with expression drawn from negative binomial distributions without spatial structure (20 replicates per sparsity level, 1,000 genes each). Two challenges are specific to this setting: the RFF features are correlated, particularly at large bandwidth scales, and zero-inflated expression vectors are heavy-tailed, violating the Gaussian assumption of standard approximations. We derive a kurtosis-corrected moment-matching formula that accounts for both effects (Methods; Supplementary Note 3; Supplementary Fig. 4).

**Fig. 3:**
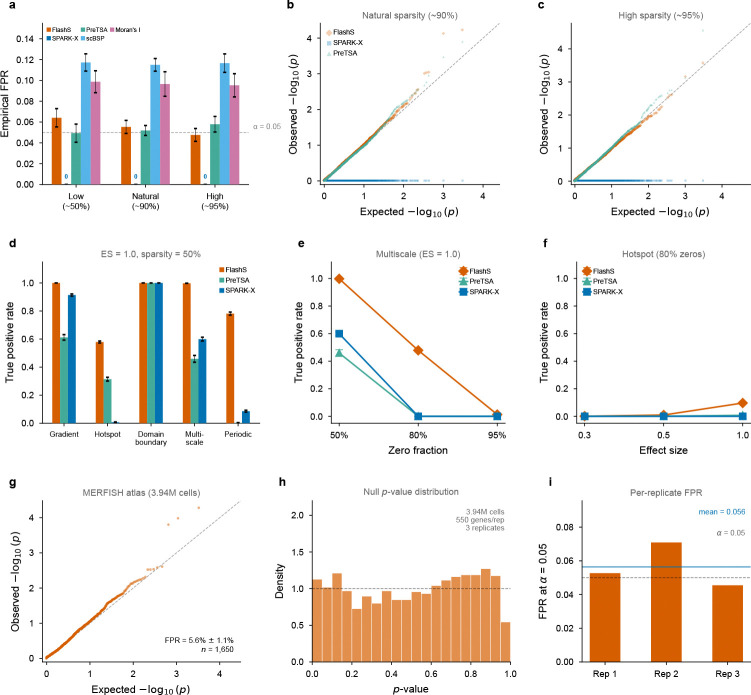
Statistical calibration and detection power. **a**, Empirical false positive rates at α=0.05 across five methods and three sparsity levels (~50%, ~90%, ~95% zeros; 20 replicates of 1,000 genes each). Dashed line marks the nominal level. SPARK-X yields FPR = 0 in all conditions (annotated). **b,c**, QQ plots of null p-values at natural (~90%) and high (~95%) sparsity (simulation). FlashS and PreTSA track the diagonal closely; SPARK-X p-values concentrate at 1 (strongly conservative). **d**, Cross-method detection power (TPR at q<0.05) across five spatial pattern types at effect size 1.0 and 50% sparsity. FlashS achieves the highest sensitivity across all pattern types; SPARK-X shows near-zero power on hotspot patterns. **e**, TPR as a function of sparsity for the multiscale pattern (effect size 1.0). At 80% zeros, FlashS retains substantial power while SPARK-X and PreTSA drop near zero. **f**, TPR as a function of effect size for the hotspot pattern at 80% sparsity. **g**, QQ plot of null p-values from global row permutation on the Allen MERFISH atlas (3.94 million cells, 550 genes, 3 replicates pooled, 1,650 null tests). FlashS maintains near-nominal FPR at million-cell scale (mean FPR = 5.6% ± 1.1%). **h**, Histogram of MERFISH null p-values with uniform reference density. **i**, Per-replicate false positive rate at α=0.05 on the MERFISH atlas; mean FPR (blue line) tracks the nominal level. Error bars in **d–f** indicate standard error across 10 replicates.

With the corrected null distribution, the empirical false positive rate at α=0.05 averages 5.6% across 60 replicates (Fig. 3a; Supplementary Fig. 3b), close to the nominal level and decreasing toward the nominal 5% at higher sparsity (4.8% at ~95% zeros). Under these simulation conditions, the q-value-based false discovery rate[13] remained below 0.1% at q<0.05 across all 60 replicates. Per-scale calibration is accurate across all bandwidth scales, including large scales where RFF features are most correlated (Supplementary Fig. 10a).

Cross-method comparison reveals a wide range of calibration quality (Fig. 3a). FlashS (4.8–6.4%) and PreTSA (4.9–5.8%) closely track the nominal level. SPARK-X yields FPR = 0 in all conditions, indicating strongly conservative inference (Fig. 3b), while scBSP (11.5–11.7%) and Moran’s I (9.5–9.9%) show inflated Type I error.

To confirm that calibration extends to atlas-scale data with realistic spatial geometry, we performed global permutation testing on the full Allen MERFISH dataset (3.94 million cells, 550 genes). Global row permutation—shuffling which cell receives which expression vector across all cells—destroys all spatial structure while preserving the real coordinate layout and per-gene marginal distributions. Across three independent replicates (1,650 total null tests), FlashS maintained a mean FPR of 5.6% ± 1.1% at α=0.05 (Fig. 3g–i), confirming calibrated inference at million-cell scale on real spatial coordinates.

As a complementary control, structured permutations that selectively preserve part of the spatial organization—within-section shuffling (preserving between-section structure) and spatial block permutation (preserving within-block autocorrelation)—yield 100% rejection across all replicates (Supplementary Table 9), consistent with the retained spatial structure at scales detectable by the multi-scale kernel. The contrast between global null (5.6% FPR) and structured null (100% rejection) indicates that detections track the degree of preserved spatial organization.

### Detection power across spatial pattern types and sparsity levels

We evaluated detection power across three spatial pattern types—hotspot (localized expression), gradient (tissue-wide trend), and periodic (oscillatory)—at varying effect sizes and sparsity levels (Supplementary Fig. 3c). Gradient patterns are detected with near-perfect power even at high sparsity, while hotspot and periodic patterns require stronger effect sizes, consistent with the challenge of detecting localized or oscillatory signals in sparse data. Sensitivity analysis confirms that the default settings (D=500, L=7) provide a robust operating point (Supplementary Fig. 1a–d).

To compare detection profiles, we simulated five canonical spatial pattern types and evaluated FlashS, SPARK-X, and PreTSA (Fig. 3d). At effect size 1.0 and 50% sparsity, FlashS achieves the highest sensitivity across all five pattern types. The advantage is largest for hotspot patterns, where SPARK-X shows near-zero power (TPR = 0.7%), and for periodic patterns, where FlashS achieves TPR = 78.2% versus 8.6% (SPARK-X) and 0.3% (PreTSA). At higher sparsity (80% zeros), FlashS retains substantial power on multiscale patterns where both SPARK-X and PreTSA drop below 1% (Fig. 3e). These detection gaps predict that parametric methods will systematically miss genes with multi-scale spatial structure in real tissue—a prediction tested in the biological analyses below.

In the following sections, we focus comparisons on two primary baselines that represent distinct methodological paradigms: SPARK-X, the strongest non-parametric kernel-based method on the benchmark (τ=0.886), and PreTSA, the leading parametric regression-based alternative (τ=0.769). Comparing FlashS against SPARK-X tests whether additional detections from a richer kernel carry biological signal; comparing against PreTSA tests whether multi-scale kernel patterns capture spatial biology that fixed-basis models miss. We first establish cross-sample reproducibility and cross-platform concordance in brain tissue, then test the simulation prediction directly in cardiac tissue, and finally assess practical completability at atlas scale.

### Cross-dataset and cross-platform generalization

**Fig. 4:**
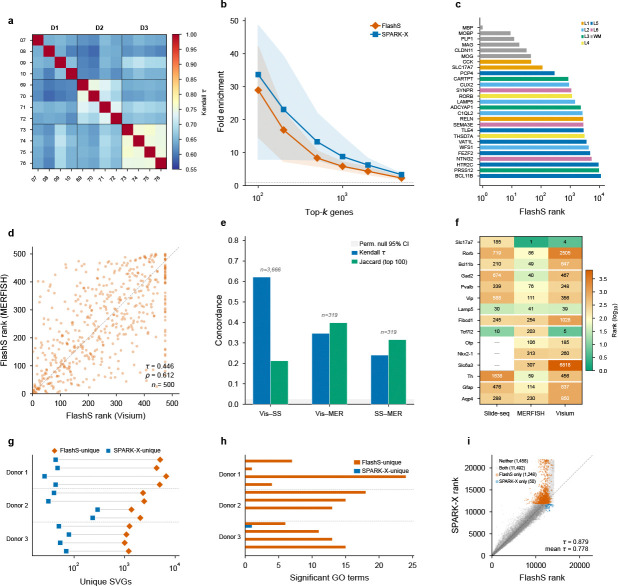
Cross-dataset and cross-platform generalization of SVG rankings. **a**, Cross-sample reproducibility of FlashS SVG rankings across 12 DLPFC Visium samples (3 donors). Same-donor pairs (diagonal blocks) show higher concordance (mean τ=0.703) than cross-donor pairs (mean τ=0.643). **b**, Fold enrichment of 27 known cortical layer markers in the top-k ranked genes, averaged across all 12 samples. Both FlashS (vermillion) and SPARK-X (blue) strongly enrich known markers. **c**, FlashS ranks of all 27 known cortical layer markers (Maynard et al. 2021), colored by assigned cortical layer. White-matter markers (MBP, MOBP, PLP1) are ranked highest, followed by layer-specific markers in order of their spatial variability. All 27 markers are detected as SVGs (q<0.05). **d**, Cross-platform SVG rank concordance between Visium mouse brain (2,702 spots) and MERFISH atlas (3.94 million cells) for 500 common genes. FlashS rankings show significant concordance (Kendall τ=0.446, Spearman ρ=0.612, permutation P<0.001). **e**, Pairwise rank concordance (Kendall τ, blue) and top-100 gene overlap (Jaccard similarity, green) across three spatial transcriptomics platforms. All pairs significantly exceed the permutation null (grey band, 95% CI). Concordance is strongest between sequencing-based platforms (Visium–Slide-seq, τ=0.605,3,666 genes) and moderate for cross-modality comparisons involving the targeted MERFISH panel (319 genes). n, number of common genes tested. **f**, Rankings of 15 established brain region markers across three platforms. Each row is a gene; columns show the rank (lower = stronger spatial signal) on Slide-seq V2, MERFISH, and Visium. Known markers are consistently ranked among the top SVGs across all three technologies. **g**, Method-specific SVG counts across all 12 DLPFC samples (log scale). Restricting to commonly tested genes with Benjamini–Hochberg correction at q<0.05, FlashS consistently detects substantially more unique SVGs (mean 2,802; vermillion) than SPARK-X (mean 84; blue). Lines connect paired counts for each sample. **h**, Number of significant Gene Ontology enrichment terms for method-specific SVGs across all 12 DLPFC samples. FlashS-unique SVGs yield 0–24 enriched terms per sample; SPARK-X-unique SVGs yield zero terms in 11 of 12 samples. **i**, Rank scatter plot comparing FlashS and SPARK-X on DLPFC sample 151673 (14,248 commonly tested genes). After Benjamini–Hochberg correction at q<0.05, FlashS detects substantially more unique SVGs than SPARK-X, with both methods showing highly concordant rankings overall.

To assess cross-sample reproducibility, we compared FlashS with SPARK-X—the strongest non-parametric baseline—on 12 Visium samples spanning the human dorsolateral prefrontal cortex (3 donors, 33,538 genes; Maynard et al.[14]). SVG rankings show high concordance across biological replicates (same-donor mean τ=0.703, cross-donor mean τ=0.643; Fig. 4a), with expected inter-individual variation exceeding technical noise. All 27 known cortical layer markers were detected as SVGs (q<0.05) by both methods, with white-matter markers (MBP, MOBP, PLP1) ranked highest, consistent with the expected depth gradient (Fig. 4c; Supplementary Fig. 8). Running SPARK-X independently on all 12 samples yielded a mean cross-method rank concordance of τ=0.778. Both methods strongly enrich known cortical markers among top-ranked genes (top-100: SPARK-X 33.7× vs FlashS 29.0×; Fig. 4b), with SPARK-X’s slightly higher enrichment at small k consistent with its more conservative detection threshold (mean 84 unique SVGs per sample versus 2,802 for FlashS).

Gene Ontology enrichment analysis of the method-specific detections shows that FlashS-unique SVGs converge on DNA-binding transcription factor activity in 7 of 12 samples, consistent with cortical layer-specific transcriptional programs, while SPARK-X-unique SVGs yield zero enriched GO terms in 11 of 12 samples (Fig. 4g,h; Supplementary Table 8). Both methods are comparably effective at detecting the strongest known spatial genes, but FlashS additionally recovers a large class of spatially organized genes—enriched for biologically coherent functions—that fall below SPARK-X’s detection threshold.

As a stringent cross-platform test, we compared SVG rankings produced independently on mouse brain datasets profiled by Visium (2,702 spots), MERFISH (3.94 million cells), and Slide-seq V2 (41,786 beads). Among 500 genes tested on both Visium and MERFISH, FlashS rankings show significant concordance (τ=0.446, ρ=0.612, permutation P<0.001; Fig. 4d), comparable to PreTSA (τ=0.460); the similar concordance across methods indicates that the strongest spatial signals replicate across platforms regardless of the detection approach. All three pairwise comparisons significantly exceed the permutation null (P<0.001; Fig. 4e,f), with known brain region markers consistently ranked among the top SVGs across all three technologies (Supplementary Fig. 6), indicating that the detected spatial signals reflect genuine biology despite order-of-magnitude differences in cell count and measurement modality.

### Biological validation and pathway-level coherence in cardiac tissue

**Fig. 5:**
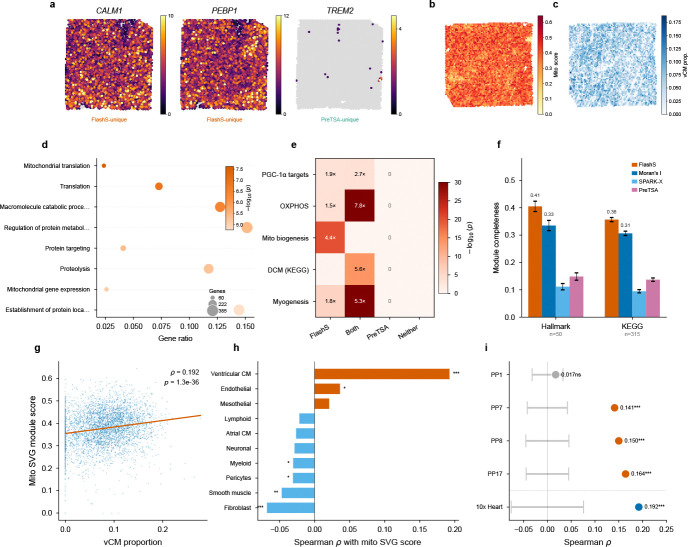
Biological validation and pathway-level coherence in human heart. **a**, Spatial expression of discordant SVGs on the Visium human heart (4,235 spots): *CALM1* (calmodulin 1) and *PEBP1* (phosphatidylethanolamine-binding protein 1; FlashS-unique, clear spatial patterns across cardiac tissue) versus *TREM2* (triggering receptor expressed on myeloid cells 2; PreTSA-unique, sparse expression in myeloid-enriched foci). Color intensity indicates raw counts; gray dots mark non-expressing spots. Full discordant gene comparison in Supplementary Fig. 7. **b**, Spatial distribution of the mitochondrial SVG module score (mean log-normalized expression of 49 curated mitochondrial biogenesis genes) across 4,235 Visium spots. **c**, Spatial distribution of deconvolved ventricular cardiomyocyte (vCM) proportion across the tissue, estimated by FlashDeconv using the Litviňuková et al. single-cell atlas. **d**, Gene Ontology enrichment of 2,669 FlashS-unique SVGs, showing spatially organized processes (mitochondrial translation, intracellular transport) missed by PreTSA. **e**, Enrichment heatmap showing hypergeometric significance ($-{log}_{10}(p)$) of four SVG categories across five curated gene sets (MSigDB). Annotations indicate fold enrichment for significant results (p<0.05); all marked results remain significant after Benjamini–Hochberg correction (q<0.05; Supplementary Table 7). FlashS-unique SVGs are strongly enriched for mitochondrial pathways; PreTSA-unique SVGs show no significant overlap with any gene set. **f**, Systematic module completeness across 365 curated pathways (50 MSigDB Hallmark, 315 KEGG) within the 14,634-gene universe. Bars show mean fraction of pathway genes detected as SVGs (q<0.05) by each method; error bars indicate s.e.m. FlashS achieves the highest completeness (mean 0.363), surpassing Moran’s I (0.310) and outperforming parametric alternatives by 2.6–3.7×. Per-pathway distributions and method-specific SVG counts in Supplementary Fig. 9. **g**, Scatter plot of vCM proportion versus mitochondrial SVG module score (ρ=0.192, $p=1.3\times 10^{-36}$). **h**, Spearman correlation of mitochondrial SVG module score with each deconvolved cell-type proportion. Ventricular cardiomyocytes show the clearest positive correlation, consistent with their high mitochondrial content. Block-permutation and gene-set leakage controls are shown in Supplementary Fig. 7. **i**, External replication on four independent healthy control Visium slides from Kuppe et al.[20] (2,043–4,269 spots per slide). Points show Spearman ρ between the transferred mito module score and cell2location-derived cardiomyocyte abundance estimates; gray bands show block-permutation null 95% CI. Three of four replication samples show significant positive association (ρ=0.14-0.16, $p_{\text{perm}}<10^{-4}$). Stars: Spearman p (***p < 0.001).

The simulation analyses predict that parametric methods will systematically miss genes whose spatial patterns span multiple scales. To test whether this translates to biologically meaningful detection differences, we compared FlashS against both anchor baselines—SPARK-X (non-parametric kernel) and PreTSA (parametric B-spline)—on a 10x Visium human heart dataset (4,235 spots, 16,716 genes; Fig. 5a). FlashS and SPARK-X show the highest pairwise rank agreement (Kendall τ=0.52, top-50 overlap 80%), consistent with both being non-parametric kernel-based approaches; both show lower agreement with PreTSA (τ=0.41 and 0.38, respectively), confirming that the parametric B-spline approach produces substantially different gene rankings. Restricting to genes tested by both methods and applying Benjamini–Hochberg correction uniformly, FlashS detects 3,699 SVGs at q<0.05 compared to 1,123 for PreTSA, with 1,030 genes detected by both methods and 93 detected only by PreTSA. Gene Ontology enrichment analysis reveals that the shared SVGs are strongly enriched for cardiac-specific functions, led by oxidative phosphorylation ($p<10^{-72}$) and aerobic respiration, consistent with the spatial organization of energy metabolism in heart tissue. FlashS-unique SVGs (2,669 genes) are enriched for additional spatially organized processes including mitochondrial translation and intracellular transport (Fig. 5d; Supplementary Table 5). In contrast, the 93 PreTSA-unique SVGs yield no significant GO terms, suggesting that these detections may be model-specific and lack support from known biological pathways. Visual inspection confirms this asymmetry: FlashS-unique SVGs such as *CALM1* and *PEBP1* show clear spatial expression patterns across the cardiac tissue, whereas top PreTSA-unique detections (e.g., *TREM2*) exhibit sparse expression limited to isolated foci (Fig. 5a; Supplementary Fig. 7).

We next asked whether this enrichment reflects a coherent biological program and whether the detected spatial patterns can be independently validated using orthogonal single-cell data.

To test this, we performed hypergeometric enrichment analysis against five curated gene sets from MSigDB[15], applying Benjamini–Hochberg correction across all 20 tests (5 gene sets × 4 SVG categories; Supplementary Table 7). FlashS-unique SVGs are significantly enriched for PGC-1α transcriptional targets (1.90-fold, odds ratio [OR] = 2.39, 95% CI 1.51–3.83, $q=1.0\times 10^{-3}$; Fig. 5e), the master regulator of mitochondrial biogenesis in cardiomyocytes[16, 17]. The dataset contains 49 curated mitochondrial biogenesis genes, spanning mitochondrial ribosomal proteins (MRPL/MRPS family), translocase complexes (TOMM/TIMM), and respiratory chain assembly factors. Of these, 40 are detected as SVGs by FlashS—39 appearing exclusively in the FlashS-only category (4.36-fold, $q=9.5\times 10^{-20}$)—with no overlap in the PreTSA-only category. This sensitivity advantage extends beyond the parametric–non-parametric divide. Moran’s I, a standard global autocorrelation statistic, detects only 28 of 49 (57%) of these genes at q<0.05 despite calling 51% of the transcriptome spatially variable genome-wide; SPARK-X detects only 9 (18%; Supplementary Table 11). The resulting detection gradient—FlashS (40) > Moran’s I (28) > SPARK-X (9) > PreTSA (1)—tracks the flexibility of each method’s spatial representation. These 40 genes form a spatially coordinated transcriptional program: they share a common upstream regulator (PGC-1*α*), are co-expressed within myocardial compartments, and their collective spatial signature—focal enrichment in ventricular regions superimposed on tissue-wide metabolic gradients—exemplifies the compound, multi-scale patterns that a fixed-degree polynomial basis is not designed to capture. This enrichment extends to disease-relevant gene sets: dilated cardiomyopathy genes (KEGG; OR = 8.81, 95% CI 5.50–14.28, $q=4.6\times 10^{-14}$ in the shared SVG category) and myogenesis hallmark genes (OR = 8.32, 95% CI 6.12–11.38, $q=2.4\times 10^{-30}$) are enriched among SVGs detected by FlashS, while PreTSA-unique SVGs show no significant overlap with any tested pathway (0 of 20 tests significant after FDR correction; Fig. 5e).

To validate the spatial pattern of these mitochondrial SVGs independently, we performed spatial deconvolution using the Litviňuková et al. human heart single-cell atlas[18] (486,134 cells and nuclei spanning 11 major cardiac cell types; 452,136 retained after our preprocessing) via FlashDeconv[19]. We then computed a per-spot mitochondrial SVG module score (mean log-normalized expression of the 49 curated mitochondrial biogenesis genes) and correlated it with deconvolved cell-type proportions across 4,235 Visium spots (Fig. 5b,c). The mitochondrial SVG score is positively correlated with ventricular cardiomyocyte proportion (Spearman ρ=0.192, $p=1.3\times 10^{-36}$; Fig. 5g), consistent with the known high mitochondrial content of ventricular cardiomyocytes, which depend on oxidative phosphorylation for continuous contractile function[18]. By contrast, atrial cardiomyocyte proportion shows no significant correlation (ρ=-0.026, p=0.085), reflecting the lower mitochondrial density in atrial tissue, while fibroblasts are negatively correlated (ρ=-0.068, $p=9.4\times 10^{-6}$; Fig. 5h). Among all 12 cell types tested, ventricular cardiomyocytes show the clearest positive association with the mitochondrial SVG pattern.

Three controls address potential confounders. First, spatial block permutation testing confirms that the vCM–mito correlation is robust to spatial dependence ($p_{\text{perm}}<10^{-4}$ across three grid resolutions; Supplementary Fig. 7). Second, gene-set leakage between the module score and deconvolution input was excluded by re-running FlashDeconv after removing all 49 mitochondrial biogenesis genes; the vCM correlation remains virtually unchanged (Δρ=0.006; Supplementary Fig. 7). Third, to test whether the mito program’s spatial signal reflects spatial structure beyond cell-type composition, we regressed out all 12 deconvolved cell-type proportions from the 16,712 tested genes prior to spatial testing. After this adjustment, 311 of 3,968 baseline SVGs remained genome-wide, and 8 of 49 curated mitochondrial biogenesis genes retained significance, indicating that the dominant source of the mito program’s spatial variation is the spatial distribution of ventricular cardiomyocytes—consistent with the deconvolution analysis. This result is expected: the spatial distribution of cell types is itself a tissue-level spatial pattern, and both FlashS and PreTSA operate on the same unadjusted expression data. The detection gap reflects the spatial model’s capacity to represent the multi-scale shape of cell-type distributions across tissue space.

To test whether this mito–cardiomyocyte association generalizes beyond a single dataset, we repeated the full analysis on four healthy control Visium slides from an independent cohort[20] (2,043–4,269 spots per slide). Despite only 16 of the 49 mito biogenesis genes being present in the Kuppe Visium panel, FlashS-unique SVGs are again significantly enriched for mitochondrial biogenesis in all 4 samples (mean fold enrichment = 2.8, hypergeometric p<0.02; Supplementary Fig. 7). The transferred 49-gene mito module score is positively associated with cell2location-derived cardiomyocyte abundance estimates in 3 of 4 samples (ρ=0.14-0.16, block permutation $p_{\text{perm}}<10^{-4}$; Fig. 5i), replicating the key finding on independent tissue from a different institution and experimental batch.

Together, these results provide convergent evidence that FlashS recovers a coherent and reproducible biological program: detection of PGC-1α-driven mitochondrial biogenesis genes, association with ventricular cardiomyocytes via single-cell deconvolution, and replication in an independent cohort. This detection gap extends beyond individual pathways: systematic module completeness analysis across 365 curated gene sets (50 MSigDB Hallmark and 315 KEGG pathways) shows that FlashS recovers a mean of 36.3% of pathway genes as SVGs across all 365 gene sets, surpassing Moran’s I (31.0%), while PreTSA and SPARK-X achieve markedly lower coverage (13.9% and 9.7%, respectively; Fig. 5f; Supplementary Table 12). A top-k controlled analysis confirms that this advantage persists after removing the confound of differing SVG counts (Supplementary Fig. 10c).

### Atlas-scale application to whole mouse brain MERFISH

**Fig. 6:**
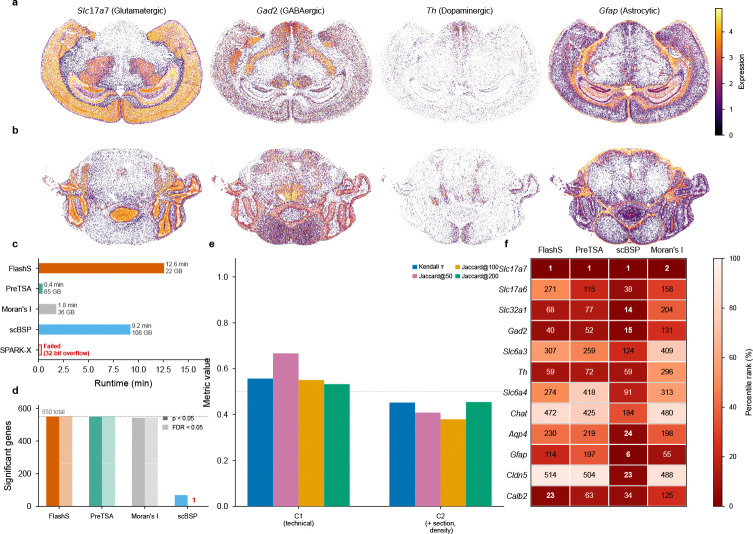
Atlas-scale SVG detection on the Allen MERFISH whole mouse brain. **a,b**, Spatial expression of four representative marker genes in mid-brain (**a**, 115,779 cells) and anterior (**b**) coronal sections: *Slc17a7* (glutamatergic, cortex), *Gad2* (GABAergic), *Th* (dopaminergic, midbrain), *Gfap* (astrocytic). Color intensity indicates log-transformed expression; gray dots mark non-expressing cells. **c**, Method scalability comparison on 3.94 million cells. PreTSA completes in 25 s with 64.7 GB; SPARK-X fails due to R’s 32-bit integer limit; scBSP requires 108 GB memory. **d**, Number of significant genes detected by each method at p<0.05 (raw) and q<0.05 (FDR-corrected). FlashS and PreTSA detect all 550 panel genes; scBSP detects only 1 gene after FDR correction. **e**, Robustness of SVG rankings to technical covariate adjustment. Bars show Kendall τ (versus unadjusted baseline) and top-k Jaccard similarity (k=50,100,200) after adjusting for log library size (C1) and section fixed effects with local cell density (C2). Moderate concordance after adjustment indicates that many spatial signals persist after controlling for technical covariates, while a subset of genes is re-ranked (Supplementary Fig. 5). **f**, Rankings of 12 known neurotransmitter system markers across methods (PreTSA ranked by F-statistic to break p-value ties). Lower rank indicates stronger spatial signal.

Spatial transcriptomics atlases now routinely profile millions of cells, yet it remains unclear which SVG detection methods can operate at this scale with calibrated inference and informative gene rankings. We applied FlashS and four competing methods to the Allen Brain Cell Atlas MERFISH dataset[21] (3,938,808 matched cells from a 500-gene panel plus 50 blank controls spanning the entire adult mouse brain; Fig. 6).

FlashS completed in 12.6 minutes with 21.5 GB peak memory, identifying all 550 matrix features as spatially variable (q<0.05). The MERFISH panel was designed to support cell-type discrimination across the atlas taxonomy[21]; because brain cell types are spatially organized, strong spatial signal is expected at this sample size. Spatial expression maps of representative markers show the known regional distributions (Fig. 6a,b).

The practical advantage of FlashS at this scale emerges from three comparisons. First, *completability*: SPARK-X failed entirely due to R’s 32-bit integer indexing limit; scBSP completed but required 108 GB peak memory—five times that of FlashS—and detected only a single gene after FDR correction (Fig. 6c,d). Second, *memory efficiency*: PreTSA completed in 25 seconds but consumed 64.7 GB, already exceeding standard workstation limits on this 550-gene panel and making whole-transcriptome analysis infeasible at this cell count. Third, *ranking resolution*: at this sample size, p-values saturate near machine precision for all methods; FlashS’s spatial effect size provides a continuous ranking metric that resolves all 550 genes, whereas PreTSA’s p-values yield only 23 distinct values (Fig. 6f). Examination of 12 established neurotransmitter system markers across 8 cell populations confirms that FlashS rankings reflect known spatial biology, with *Slc17a7* (glutamatergic) ranked first (see Methods for full marker list).

We assessed the robustness of these rankings to technical covariates by regressing out log library size (C1) and section fixed effects with local cell density (C2) prior to spatial testing. Technical adjustment preserved the overall scale of the effect sizes (median attenuation ratio 1.11; Fig. 6e) but produced moderate reordering of gene ranks (C1: τ=0.56;C2:τ=0.45), indicating that many high-signal genes remain robust while a subset is meaningfully re-ranked. Stratified analyses within individual brain sections (mean τ=0.53) and within single cell types (mean τ=0.41; Supplementary Fig. 5) show that spatial patterns persist within homogeneous subpopulations.

## Discussion

The central finding of this work is that the choice of spatial model in SVG detection has direct consequences for which biological programs are discovered. Across two tissues and two methodologically distinct comparators, a consistent pattern emerges: FlashS-unique SVGs converge on tissue-appropriate pathways—mitochondrial biogenesis in heart, transcription factor activity in cortex—while competitor-unique detections lack coherent enrichment. That this asymmetry extends to genome-wide module completeness across 365 pathways and replicates in an independent cohort argues that the detection gap reflects genuine spatial biology, not method-specific noise.

The mechanism underlying this detection advantage is representational. Sampling hundreds of stochastic frequencies from a Gaussian kernel’s spectral density creates a richer spatial basis than the fixed, low-rank projections of existing scalable methods: SPARK-X’s periodic cosine transforms each probe a single spatial frequency, while PreTSA’s B-spline basis spans a fixed polynomial space whose resolution is determined by knot placement. Both approaches achieve tractability by constraining the class of detectable patterns, and the benchmark and real-tissue analyses confirm that these constraints translate to measurable detection differences (Figs. 3d, 5e,f). A distinct spectral route enters spatial transcriptomics through graph signal processing (SpaGFT[22]), but on the SVG benchmark the two spectral approaches yield markedly different accuracy (Fig. 2a), reflecting the distinction between discrete graph eigenbases and continuous kernel spectral sampling with universal approximation guarantees.

Cross-platform concordance reinforces this view: SVG rankings replicate across Visium, Slide-seq V2, and MERFISH despite order-of-magnitude differences in cell count and fundamentally different measurement modalities (Fig. 4d–f). For practitioners, the implication is concrete: the choice of spatial model can determine which functional programs are prioritized for downstream validation, and this choice has been invisible until systematic cross-method comparison.

Three design choices complement the kernel representation: operating on raw counts preserves spatial signal that library-size normalization can attenuate (Supplementary Table 6); the three-part test prevents worst-case failures on datasets where any single channel underperforms (Supplementary Fig. 1e–i); and the binary and rank channels confer robustness to library-size confounding, as confirmed by the covariate adjustment analysis on the MERFISH atlas (Supplementary Fig. 5c,d).

At atlas scale, memory efficiency becomes the binding constraint. FlashS’s per-gene working memory of O(D)—independent of cell count—enables million-cell analysis on standard workstations, whereas methods with O(n⋅g) memory scaling become impractical beyond ~200,000 cells without high-memory infrastructure. When p-values saturate at machine precision, as occurs for all methods at extreme sample sizes, a continuous effect-size metric preserves meaningful gene ordering for biological prioritization. We note that PreTSA achieves faster wall-clock time at all tested scales through shared dense matrix operations; FlashS’s primary advantage lies in detection accuracy and memory efficiency, and on typical Visium-scale datasets it completes in 1–2 minutes, adequate for routine analysis.

Several limitations warrant discussion. The Open Problems benchmark generates spatial patterns via Matérn Gaussian process smooths; although multiple lines of evidence argue against systematic bias toward kernel detectors—classical GP methods perform poorly (τ<0.68), FlashS uses a different kernel family, and the alpha-mixing metric is model-agnostic—benchmarks incorporating diverse generative models would further strengthen evaluation. The kurtosis-corrected Satterthwaite approximation brings false positive rates close to nominal across sparsity levels, but two-parameter moment matching cannot fully capture higher-order tail behavior of zero-inflated projections; saddlepoint approximations could tighten calibration at increased computational cost. The Gaussian kernel is stationary, precluding spatially adaptive detection resolution, though the multi-scale bandwidth ensemble partially mitigates this. The method has been validated on two-dimensional data; performance on three-dimensional tissue architectures from emerging volumetric technologies remains to be characterized. Finally, FlashS identifies globally spatially variable genes but does not perform spatial domain detection or cell-type-specific spatial testing, which are complementary analytical tasks.

The frequency-domain framework opens natural extensions. Non-Gaussian spectral densities such as spectral mixture kernels[23] could improve detection of oscillatory patterns—the RFF sampling mechanism makes kernel replacement straightforward, though adapting mixture hyperparameters to the moment-based null distribution remains an open problem. Incorporating spatial covariates would enable conditional SVG detection, and generalization to multivariate statistics could identify co-varying gene programs beyond univariate spatial variability. As spatial atlases scale to whole organs and organisms, the principle that spectral transformations unlock sparsity-aware kernel computation may find application across the broader landscape of high-throughput spatial biology.

## Methods

### Random Fourier Feature representation of spatial coordinates

To test for spatial variation without constructing n×n distance matrices, FlashS employs Random Fourier Features (RFF)[9]. By Bochner’s theorem, shift-invariant positive definite kernels can be represented as Fourier transforms of probability measures (Supplementary Note 1).

For each spatial location $s_{i}\in R^{2}$, we construct a D-dimensional feature vector:

(1) $$z\left(s_{i}\right)=\sqrt{\frac{2}{D}}{\left[cos\left(\omega_{1}^{⊤}s_{i}+b_{1}\right),\ldots ,cos\left(\omega_{D}^{⊤}s_{i}+b_{D}\right)\right]}^{⊤}$$

where $\omega_{k}∼𝒩\left(0,\sigma^{-2}I\right)$ are frequency vectors sampled from the spectral density of a Gaussian kernel with bandwidth σ, and $b_{k}∼Uniform(0,2\pi )$ are phase offsets. The inner product $\left\langle z(s),z\left(s^{\prime }\right)\right\rangle$ approximates the Gaussian kernel $k\left(s,s^{\prime }\right)=exp(-{\left\|s-s^{\prime }\right\|}^{2}/2\sigma^{2})$.

### Adaptive multi-scale bandwidth selection

Spatial expression patterns in tissue span multiple characteristic scales, from cellular neighborhoods to tissue-wide gradients. Rather than using fixed quantile-based bandwidths, FlashS employs adaptive bandwidth selection based on intrinsic geometric properties of the spatial point cloud. The local scale is set by the median nearest-neighbor distance, capturing fine-grained structure. The global scale is estimated from the Fiedler value[24] (second smallest eigenvalue of the graph Laplacian), providing the characteristic length scale $\ell_{\text{spectral}}=1/\sqrt{\lambda_{2}}$ for tissue-wide gradients.

FlashS generates L (default 7) bandwidth values log-spaced between local and global scales. The D random features are distributed across scales via divmod (D,L): each scale receives ⌊D/L⌋ features, with the first D mod L scales receiving one extra.

### SVG detection via sparse sketching

For each gene, FlashS tests whether standardized expression y associates with spatial features Z. The test statistic is:

(2) $$T={\left\|y^{⊤}Z\right\|}_{2}^{2}=\sum_{k=1}^{D}{\left(\sum_{i=1}^{n}\;y_{i}z_{ik}\right)}^{2}$$

The key computational strategy is computing this statistic without materializing the n×D feature matrix Z, leveraging sparse sketching[25] (Supplementary Note 2). For sparse expression vectors with nnz non-zero entries:

(3) $$v_{k}=\sqrt{\frac{2}{D}}\sum_{i:y_{i}\neq 0}y_{i}\;cos\left(\omega_{k}^{⊤}s_{i}+b_{k}\right)$$

achieving O(nnz⋅D) per gene. Centering is performed implicitly by pre-computing the column sums $1^{⊤}Z$ once during model fitting and subtracting $\overset{‾}{y}\left(1^{⊤}Z\right)$ from the sparse projection, preserving sparsity (Supplementary Note 2). The centering vector $1^{⊤}Z$ is computed exactly in one O(nD) pass (with O(D) memory) rather than subsampled. This is required for calibration: centering error enters the quadratic statistic as ${\left\|\overset{‾}{y}\delta \left(1^{⊤}Z\right)\right\|}^{2}$, which can dominate the null scale when n is much larger than the subsample size used for moment estimation.

### Three-part test for zero-inflated spatial data

To handle zero-inflation, FlashS applies three complementary test channels per gene and per bandwidth scale:

1. **Binary test**: Tests whether the indicator 1(y>0) shows spatial structure, capturing patterns in gene detection.
2. **Rank test**: Tests whether rank-transformed non-zero values vary spatially, providing outlier-robust intensity assessment.
3. **Direct test**: Tests whether raw expression values show spatial structure, preserving library-size-correlated signal.

In addition to the multi-scale RFF kernels, FlashS includes a linear spatial trend test for each channel, regressing the (binary, rank, or direct) expression vector onto centered spatial coordinates. This test detects tissue-wide linear gradients that may fall between Gaussian bandwidth scales. The three resulting p-values ($\chi_{d}^{2}$ under the null, d=spatialdimensionality) enter the Cauchy combination alongside the RFF-based p-values.

### Cauchy combination across scales and test types

Per-scale p-values from all three test types are combined via the Cauchy combination test[10]:

(4) $$T_{CCT}=\frac{1}{K}\sum_{j=1}^{K}\tan\left[\left(\frac{1}{2}-p_{j}\right)\pi \right]$$

where K=3L+3: three test channels across L bandwidth scales, plus three linear spatial trend tests. Under the null, $T_{CCT}$ follows a standard Cauchy distribution regardless of the dependency structure among the K component tests, requiring only that each marginal p-value is valid under the null; this yields a single combined p-value per gene.

### Null distribution and p-value computation

Under the null hypothesis of no spatial variation, the test statistic $T=\sum_{k}v_{k}^{2}$ where $v_{k}=y^{⊤}z_{k}$ (centered). Since the RFF columns $z_{k}$ are correlated—particularly at large bandwidth scales where multiple cosine features approximate similar low-frequency polynomials—the standard Satterthwaite approximation that assumes independent $v_{k}$ underestimates the null variance. Specifically, for jointly normal $v_{k},Var[T]=2{\left\|\Sigma_{v}\right\|}_{F}^{2}$ where $\Sigma_{v}=\sigma_{y}^{2}Z_{c}^{⊤}Z_{c}/n$ is the covariance of the projection vector, and $Z_{c}$ denotes the column-centered feature matrix.

We use a kurtosis-corrected Satterthwaite moment matching[26] with $E\left[T_{\ell }\right]=n\sigma_{y}^{2}\sum_{k}Var\left[z_{k,\ell }\right]$ and the per-scale variance:

$$Var\left[T_{\ell }\right]=2\sigma_{y}^{4}n^{2}{\left\|\Sigma_{z,\ell }\right\|}_{F}^{2}+\kappa_{4}\sigma_{y}^{4}\sum_{i=1}^{n}{\left\|z_{c,\ell ,i}\right\|}^{4}$$

where $\Sigma_{z,\ell }$ is the centered feature covariance within scale ℓ and $\kappa_{4}=E[{\left(\tilde{y}/\sigma_{y}\right)}^{4}]-3$ is the per-gene excess kurtosis[27]. The second term—a kurtosis correction not present in standard quadratic-form approximations used by existing SVG methods—accounts for the leptokurtic distribution of zero-inflated expression vectors ($\kappa_{4}\gg 0$), which causes the Gaussian-only formula to underestimate the null variance and inflate p-values (Supplementary Note 3). Both terms are estimated once on a coordinate subsample (M=10,000 by default) at $O\left(M\sum_{\ell =1}^{L}\;D_{\ell }^{2}\right)$ cost. The scale and degrees of freedom of the approximating $\kappa \chi_{\nu }^{2}$ distribution are then $\kappa =Var\left[T_{\ell }\right]/\left(2E\left[T_{\ell }\right]\right)$ and $\nu =2E{\left[T_{\ell }\right]}^{2}/Var\left[T_{\ell }\right]$. This provides analytic p-values without permutation (see Results for FPR and FDR assessment).

### Multiple testing correction

P-values across genes are adjusted using the Benjamini–Hochberg procedure to control the false discovery rate at level α (default 0.05). This ensures a unified correction framework across all methods in comparative analyses.

### Benchmarking

We evaluated FlashS on the Open Problems benchmark for SVG detection[11], which includes 11 published SVG detection methods on its leaderboard (see Results for dataset description). All leaderboard methods were scored with default parameters as specified in the benchmark.

Each method produces a per-gene spatial variability score; the benchmark evaluates methods by Kendall τ between predicted and ground truth scores. As in the Open Problems framework, each method uses the score most appropriate to its statistical model: SpatialDE, SpatialDE2, and SOMDE report the fraction of spatial variance (FSV); Moran’s I reports the I statistic; SpaGFT and Spanve report method-specific test statistics; scGCO uses $-{log}_{10}(p)$. FlashS uses the spatial effect size, defined as the maximum ratio of observed to expected test statistic across the three test channels (binary, rank, direct); an ablation across all 50 datasets confirms that $-{log}_{10}(p)$. alone yields substantially lower concordance (τ=0.868), as p-values saturate at moderate sample sizes, whereas the effect size provides a continuous, non-saturating ranking metric (Supplementary Table 13).

FlashS was applied to raw counts without library-size normalization or log-transformation, using D=500 random features, L=7 bandwidth scales, and the three-part test (binary, rank, direct). Three additional methods not included in the original leaderboard—PreTSA[8], Hotspot[7], and scBSP[6]—were benchmarked on the same 50 datasets using their default parameters. PreTSA was run using its Python implementation with default knot setting (knot = 0, corresponding to a cubic B-spline basis without internal knots) on library-size-normalized and log-transformed data as specified by the authors; PreTSA also provides an automatic knot selection mode, but we used the default configuration as recommended in the package documentation. All benchmarks were performed on compute nodes of a shared HPC cluster (AMD EPYC, 16 cores). FlashS version 0.1.0 was used with Python 3.11, NumPy 1.26, SciPy 1.12, and Numba 0.59.

### Scalability experiments

Scalability was evaluated on simulated datasets with 5,000 genes and cell counts from 1,000 to 1,000,000 (3 replicates per condition). Spatial coordinates were sampled uniformly; expression was drawn from a negative binomial distribution (n=1, p=0.1; ~90% zeros). Runtime was measured as wall-clock time from model fitting through p-value computation; peak memory was recorded via the resource module.

Runtime comparison against nine SVG detection methods—Moran’s I (Squidpy[28]), SpatialDE[1], Hotspot[7], SPARK-X[5], SOMDE[29], scBSP[6], Spanve[30], nnSVG[4], and PreTSA[8]—was performed at cell counts up to 50,000 (3 replicates each; 2-hour timeout, 50 GB memory limit per run). SPARK-X and nnSVG were called via rpy2 from their R implementations. Extended FlashS–PreTSA comparison was performed up to 1,000,000 cells (Supplementary Table 4). All experiments were performed on 16-core compute nodes (AMD EPYC).

### Atlas-scale MERFISH analysis

We applied FlashS, PreTSA, SPARK-X, scBSP, and Moran’s I (Squidpy) to the Allen Brain Cell Atlas MERFISH dataset[21] (specimen C57BL6J-638850). The processed expression matrix contains 4,334,174 cells and 550 rows corresponding to a 500-gene panel plus 50 blank controls; after matching cell identifiers between the expression matrix (H5AD format) and spatial metadata, 3,938,808 cells were retained. Expression data were used as raw counts without normalization.

FlashS was run with default parameters (D=500, L=7). SPARK-X was called via rpy2 from its R implementation; scBSP (v0.3.1) via its Python interface; Moran’s I via Squidpy (k=6 nearest neighbors). PreTSA was run on library-size-normalized and log-transformed data; because p-values saturate at machine zero at this sample size, gene rankings were derived from the F-statistic. Each method was run as an isolated job on a 16-core compute node with 128 GB memory allocation.

Biological validation used 12 established neurotransmitter system markers: *Slc17a7* and *Slc17a6* (glutamatergic), *Slc32a1* and *Gad2* (GABAergic), *Slc6a3* and *Th* (dopaminergic), *Slc6a4* (serotonergic), *Chat* (cholinergic), *Aqp4* and *Gfap* (astrocytic), *Cldn5* (endothelial), and *Calb2* (interneuron).

### Structured null testing on multi-section atlas data

To characterize rejection behavior under structured null hypotheses that preserve part of the spatial organization, we applied three permutation strategies to the full Allen MERFISH atlas (3,938,808 cells, 550 genes) following the exchangeability-block framework[31]: (i) global row permutation (N0), which shuffles expression vectors across all cells, destroying all spatial structure; (ii) within-section permutation (N1), which shuffles expression only among cells within the same brain section, preserving between-section spatial structure; and (iii) spatial block permutation (N2), which partitions each section into a 6 × 6 grid and permutes expression vectors between grid blocks, preserving within-block spatial autocorrelation. For N0, 3 independent replicates of 550 genes were run (1,650 null tests); for N1, 10 replicates (5,500 tests); for N2, 6 replicates (3,300 tests). The rejection rate was computed as the fraction of genes with p<0.05; for N0, where all spatial structure is destroyed, this corresponds to the empirical false positive rate (Supplementary Table 9).

### Confounding sensitivity analysis

To test whether SVG rankings on the MERFISH atlas are driven by technical or compositional confounders, we applied FlashS to the same 3,938,808 cells after progressively regressing out potential confounders. Covariate sets were defined as: C0 (no adjustment; baseline), C1 (technical: log total counts; no mitochondrial genes are present in the 550-gene MERFISH panel), and C2 (C1 + brain-section fixed effects + local cell density). Cell density was computed per section via 2D histogram binning (50 × 50 grid). Residualization used Frisch–Waugh–Lovell demeaning for the categorical fixed effect (section), followed by ordinary least squares projection for continuous covariates (log total counts, cell density). FlashS was then applied to the residualized expression matrix with min_expressed = 0 (since residuals are no longer sparse). Stability was assessed by Kendall τ and top-*k* Jaccard between each adjusted ranking and the C0 baseline. Stratified analyses ran FlashS independently within the 5 largest brain sections and 5 most abundant cell-type classes, comparing within-stratum rankings to the C0 baseline.

### Simulation studies

For Type I error assessment, null datasets were generated on a 50 × 50 grid (2,500 spots) at three zero-fraction levels: ~50% (Poisson, λ=0.7), ~90% (negative binomial, n=1, p=0.9), and ~95% (negative binomial with additional 50% dropout). Expression was drawn without spatial structure (20 replicates of 1,000 genes each, yielding 60,000 null tests). For power evaluation, datasets were generated on the same grid with 500 spatially variable and 500 null genes per replicate (10 replicates), testing hotspot (localized Gaussian envelope), gradient (linear expression ramp), and periodic (sinusoidal) patterns at effect sizes 0.1–0.5 and sparsity levels 10–50%.

For cross-method power comparison (Fig. 3d–f), five spatial pattern types were simulated: gradient, hotspot, domain boundary (sharp step function), multiscale (gradient + hotspot superposition), and periodic (sinusoidal). Each replicate contained 200 SVGs and 200 null genes (10 replicates) at effect sizes 0.3, 0.5, and 1.0 and sparsity levels 50%, 80%, and 95%. Background expression was drawn from a negative binomial distribution. FlashS, SPARK-X, and PreTSA were each applied with default parameters. Ablation experiments varied D∈{50,100,200,500,1,000} (with L=7 fixed) and L∈{1,3,5,7,10} (with D=500 fixed), with 5 replicates per condition.

### DLPFC cross-dataset validation

We applied FlashS and SPARK-X to 12 Visium samples from the Human DLPFC dataset[14] (3 donors, 4 samples each; 33,538 genes per sample). FlashS was run with default parameters on raw counts. SPARK-X was run independently on all 12 samples via rpy2 for cross-method comparison. Cross-sample consistency was assessed by pairwise Kendall τ correlation of gene effect-size rankings. Marker enrichment was evaluated against 27 known cortical layer markers curated from Maynard et al. (spanning layers L1–L6 and white matter). Fold enrichment at top-k was computed as the fraction of markers in the top k genes divided by the expected fraction under random ranking.

### Cross-platform SVG validation

To assess cross-platform generalization, we compared SVG rankings on three independently profiled mouse brain datasets: 10x Visium (2,702 spots, 32,285 genes), Allen MERFISH atlas (3,938,808 cells, 550 genes), and Slide-seq V2 hippocampus (41,786 beads, 4,000 highly variable genes). Rankings were computed independently per platform using default parameters. Cross-platform concordance was assessed by Kendall τ, Spearman ρ, and top-k Jaccard similarity on common genes, with significance evaluated via 1,000-replicate permutation tests.

### Gene Ontology enrichment analysis

Gene Ontology enrichment of method-specific SVGs was performed using g:Profiler[32] (GO:BP, GO:MF, GO:CC, KEGG, Reactome; g:SCS threshold p<0.05) with commonly tested genes as background. For the heart dataset, the 14,634 genes tested by both FlashS and PreTSA formed the background. For DLPFC, per-sample common gene universes were used for FlashS–SPARK-X comparison across 12 samples. Genes were categorized by method-specific detection at q<0.05 (Benjamini–Hochberg).

### Heart metabolic pathway enrichment and deconvolution validation

Hypergeometric enrichment analysis was performed against five curated gene sets from MSigDB[15]: PPARGC1A_TARGET_GENES (78 of 83 genes in universe), HALLMARK_OXIDATIVE_PHOSPHORYLATION (199 genes), a curated mitochondrial biogenesis set (49 genes spanning MRPL/MRPS, TOMM/TIMM, and assembly factors), KEGG_DILATED_CARDIOMYOPATHY (71 genes), and HALLMARK_MYOGENESIS (177 genes). The 14,634 genes tested by both methods served as background. Genes were stratified into four categories based on FlashS and PreTSA detection at q<0.05 (Benjamini–Hochberg).

Spatial deconvolution used the Litviňuková et al. single-cell atlas[18] (486,134 cells; 452,136 retained across 12 cell types) via FlashDeconv[19] with default parameters (sketch dimension 512, 2,000 HVGs). A per-spot mitochondrial SVG module score (mean log_1p_-normalized expression of the 49 curated genes) was correlated with deconvolved cell-type proportions via Spearman rank correlation. Moran’s I (Squidpy, k=6) and SPARK-X were run for comparison on the same dataset.

Spatial block permutation testing (mϵ{6,8,10}, 10,000 permutations) accounted for spatial autocorrelation. Gene-set leakage was excluded by re-running deconvolution after removing the 49 mitochondrial biogenesis genes from the expression matrix. Module completeness across 365 curated gene sets (50 MSigDB Hallmark 2020, 315 KEGG 2021 Human) was defined as $\left|G\cap {SVG}_{M}\cap U\right|/|G\cap U|$ for each gene set G and method M within the 14,634-gene universe; gene sets with fewer than 5 genes in U were excluded.

### External replication on independent cardiac cohort

Replication used four healthy control Visium slides from Kuppe et al.[20] (patients P1, P7, P8, P17; 2,043–4,269 spots per slide, 13,144–15,730 genes after filtering). FlashS and PreTSA were run with identical parameters as the discovery analysis. Pre-computed cell2location[33] deconvolution proportions provided in the source data were used directly; because all four slides are from the left ventricle, the cardiomyocyte proportion serves as a ventricular cardiomyocyte proxy. The transferred 49-gene mito module score and spatial block permutation tests followed the same protocol as the discovery analysis. Only 16 of 49 mitochondrial biogenesis genes were present in the Kuppe Visium gene panel.

### Implementation

FlashS is implemented in Python using NumPy and SciPy for linear algebra and Numba[34] for JIT-compiled parallel kernels. Key optimizations include sparse sketching for O(nnz⋅D) projection (with full per-gene complexity O(nnz⋅D+nnz·lognnz) including rank transformation), implicit centering to preserve sparsity, and a fused triple projection kernel that evaluates all three test types in a single pass over the non-zero entries. Default parameters: D=500 random features, L=7 bandwidth scales. Source code is available at https://github.com/cafferychen777/FlashS. Model fitting uses one exact O(nD) centering pass plus subsampled moment estimation for variance terms; this keeps inference calibrated while preserving scalability in the gene-wise testing stage.

## Supplementary Material

Supplement 1

Supplementary information

Supplementary information is available for this paper.

## Data Availability

The Allen Brain Cell Atlas MERFISH data are publicly available at https://portal.brain-map.org/atlases-and-data/bkp/abc-atlas. The Visium human heart dataset is publicly available from 10x Genomics (https://www.10xgenomics.com/datasets/human-heart). The Human DLPFC Visium data[14] are available via the spatialLIBD Bioconductor package (http://spatial.libd.org/spatialLIBD/). The Slide-seq V2 hippocampus dataset[35] is available from the Broad Institute Single Cell Portal. Open Problems benchmark datasets are available through the Open Problems framework[11]. The Kuppe et al. healthy control Visium slides[20] are available from Zenodo (https://zenodo.org/records/6578047).

## Code availability:

FlashS is freely available at https://github.com/cafferychen777/FlashS under the MIT license.
